# CRISPR-Cas immunity is repressed by the LysR-type transcriptional regulator PigU

**DOI:** 10.1093/nar/gkad1165

**Published:** 2023-12-07

**Authors:** Leah M Smith, Hannah G Hampton, Mariya S Yevstigneyeva, Marina Mahler, Zacharie S M Paquet, Peter C Fineran

**Affiliations:** Department of Microbiology and Immunology, University of Otago, PO Box 56, Dunedin 9054, New Zealand; Genetics Otago, University of Otago, PO Box 56, Dunedin 9054, New Zealand; Maurice Wilkins Centre for Molecular Biodiscovery, University of Otago, PO Box 56, Dunedin 9054, New Zealand; Department of Microbiology and Immunology, University of Otago, PO Box 56, Dunedin 9054, New Zealand; Department of Microbiology and Immunology, University of Otago, PO Box 56, Dunedin 9054, New Zealand; Department of Microbiology and Immunology, University of Otago, PO Box 56, Dunedin 9054, New Zealand; Genetics Otago, University of Otago, PO Box 56, Dunedin 9054, New Zealand; Maurice Wilkins Centre for Molecular Biodiscovery, University of Otago, PO Box 56, Dunedin 9054, New Zealand; Department of Microbiology and Immunology, University of Otago, PO Box 56, Dunedin 9054, New Zealand; Laboratory of Microbiology, Department of Agrotechnology and Food Sciences, Wageningen University, Dreijenplein 10, 6703 HB Wageningen, The Netherlands; Department of Microbiology and Immunology, University of Otago, PO Box 56, Dunedin 9054, New Zealand; Genetics Otago, University of Otago, PO Box 56, Dunedin 9054, New Zealand; Bioprotection Aotearoa, University of Otago, PO Box 56, Dunedin 9054, New Zealand; Maurice Wilkins Centre for Molecular Biodiscovery, University of Otago, PO Box 56, Dunedin 9054, New Zealand

## Abstract

Bacteria protect themselves from infection by bacteriophages (phages) using different defence systems, such as CRISPR-Cas. Although CRISPR-Cas provides phage resistance, fitness costs are incurred, such as through autoimmunity. CRISPR-Cas regulation can optimise defence and minimise these costs. We recently developed a genome-wide functional genomics approach (SorTn-seq) for high-throughput discovery of regulators of bacterial gene expression. Here, we applied SorTn-seq to identify loci influencing expression of the two type III-A *Serratia* CRISPR arrays. Multiple genes affected CRISPR expression, including those involved in outer membrane and lipopolysaccharide synthesis. By comparing loci affecting type III CRISPR arrays and *cas* operon expression, we identified PigU (LrhA) as a repressor that co-ordinately controls both arrays and *cas* genes. By repressing type III-A CRISPR-Cas expression, PigU shuts off CRISPR-Cas interference against plasmids and phages. PigU also represses interference and CRISPR adaptation by the type I-F system, which is also present in *Serratia*. RNA sequencing demonstrated that PigU is a global regulator that controls secondary metabolite production and motility, in addition to CRISPR-Cas immunity. Increased PigU also resulted in elevated expression of three *Serratia* prophages, indicating their likely induction upon sensing PigU-induced cellular changes. In summary, PigU is a major regulator of CRISPR-Cas immunity in *Serratia*.

## Graphical Abstract

**Graphical Abstract:**
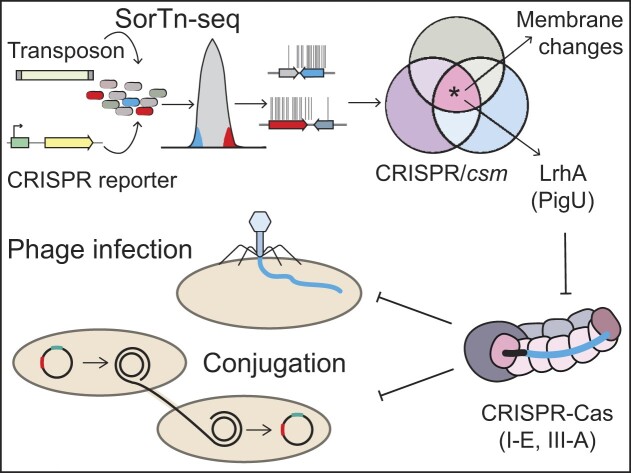

## Introduction

In almost every environment bacteria are outnumbered by their viral invaders – bacteriophages (phages). Indeed, there are an estimated 10^31^ phages on Earth that cause ∼10^25^ infections of bacteria every second (1,2). These phage-bacterium interactions influence bacterial evolution and nutrient cycling (3–5). To protect themselves, bacteria have evolved many defence strategies, including diverse innate immune mechanisms and CRISPR-Cas adaptive immunity (6,7). CRISPR-Cas systems are small RNA-based defence mechanisms. CRISPR ‘memory banks’ contain short repeats separated by similar-sized ‘spacers’ that contain genetic memories of past infections. Immunity involves three phases. During adaptation short invader-derived sequences are added as new spacers to the CRISPR array. Next, expression results in a precursor CRISPR RNA (crRNA) and its processing into small guide crRNAs by Cas proteins. Finally, Cas protein(s) and the crRNAs form interference complexes that recognise and degrade complementary nucleic acids. Although many CRISPR-Cas systems share similarities, there are two major classes (Class 1 and 2) made up of six major types (I–IV), which are further divided into >30 sub-types (8). Class 1 systems are composed of systems encoding multi-subunit interference complexes, such as type I systems, which target DNA, and type III systems, which target both DNA and RNA in a transcription-dependent manner (8). Type III systems also encode accessory nucleases which often result in collateral DNA/RNA damage (9). This diversity provides a bounty of proteins to exploit as biotechnological tools (10).

Despite the obvious immune benefits to bacteria and archaea that contain CRISPR-Cas systems, there are downsides to harbouring these systems. For example, CRISPR-Cas systems can make mistakes while recognising invaders and may incorrectly generate immunity against their own host genomes, sometimes resulting in self-targeting and cell death (i.e. autoimmunity) (11–15). Indeed, CRISPR-Cas systems are proposed to incur a fitness cost to cells (16–18). Therefore, in the absence of invaders, it is vital for bacteria to limit CRISPR-Cas activity and the potential costs. Conversely, when exposed to invaders, an elevated immune response is desirable (19,20).

While there have been considerable advances in deciphering adaptation, processing and interference, regulation of CRISPR-Cas expression is less well understood (19,20). One conserved form of CRISPR-Cas regulation is through quorum sensing (QS), a widespread form of cell-cell communication in which accumulation of extracellular chemical signals can alter population gene expression (21). QS upregulates adaptive immunity at high cell density when the risk of a phage epidemic is increased (22,23). The progress of identifying mediators of CRISPR-Cas activity had been hindered by the lack of unbiased discovery methods. Recently, to overcome this limitation, we developed SorTn-seq (24,25). SorTn-seq is a high throughput method that couples high-density random transposon mutagenesis with fluorescent reporter genes, fluorescence activated cell sorting (FACS) and transposon insertion deep-sequencing to identify regulators of any gene of interest.

We previously applied SorTn-seq to discover regulators of the type III-A *cas* (*csm*) operon in *Serratia* sp. ATCC39006 strain LacA (hereafter *Serratia*) (24,25). This *Serratia* strain is an environmental isolate that encodes type I-E, I-F and III-A CRISPR-Cas systems, in addition to multiple toxin-antitoxin and other defence systems (22,26). Using SorTn-seq, we identified and characterised an Rcs stress response pathway that inversely controls CRISPR-Cas and surface immunity. This approach also allowed a thorough genome-wide identification of the regulators of the type III-A *cas* operon. However, we did not know whether the CRISPR array promoters associated with the III-A system (CRISPR3 and CRISPR4) had coordinate or separate regulation compared with the *cas* operon. In addition, no studies have systematically assessed genes that influence CRISPR array expression. Here, we identified numerous genetic loci affecting CRISPR array expression for the type III-A system in *Serratia* and compared these to genes controlling the type III-A *cas* operon. We further characterised a key LysR-type transcriptional regulator called PigU (a homologue of *Escherichia coli* LrhA) that co-ordinately repressed type III-A CRISPR array and *cas* gene expression and hence CRISPR-Cas interference activity. PigU also repressed interference and adaptation by the type I-F system.

## Materials and methods

### Bacterial strains, bacteriophages, plasmids and culture conditions

Bacterial strains and plasmids used in this study are outlined in Supplementary Tables S1 and S2, respectively. Oligonucleotides are listed in Supplementary Table S3. *Serratia sp*. ATCC 39006 LacA and *Escherichia coli* strains were grown at 30°C and 37°C, respectively, in Lysogeny Broth (LB) with shaking at 180 rpm or on 1.5% (w/v) LB-agar (LBA) plates. When required, antibiotics and supplements were added to the media, including ampicillin (Ap, 100 μg/ml), chloramphenicol (Cm, 25 μg/ml), kanamycin (Km, 50 μg/ml), tetracycline (Tc, 10 μg/ml), isopropyl β-D-1-thiogalactopyranoside (IPTG, 100 μM), arabinose (0.02% w/v), glucose (0.2% w/v) and aminolevulinic acid (ALA, 50 μg/ml). Plasmids were transformed into *E. coli* ST18 or DH5α via heat shock and moved into *Serratia* via conjugation using *E. coli* ST18 as the donor. All plasmids and strains were confirmed by Sanger sequencing. Bacterial density was measured using a Jenway 6300 spectrophotometer at 600 nm (OD_600_). Bacteria were stored in 50% (v/v) glycerol at − 80°C. Bacteriophages ϕOT8 (27) and JS26 (28) was stored in phage buffer (10 mM Tris Base (pH 7.4); 10 mM MgSO_4_; 0.01% (w/v) gelatin) at 4°C. Generalised transduction to create double mutant strains was performed with ϕOT8 as described previously (29).

### Construction of type III-A CRISPR-cas phage JS26 targeting strain

To introduce a type III-A phage JS26-targeting spacer into the *Serratia* chromosome, the native CRISPR3 array was replaced with a mini-array (repeat-spacer-repeat) using allelic exchange mutagenesis as previously described (25) with pPF3581 in the WT (LacA) background. The resulting strain (PCF925) contains one spacer targeting the phage JS26 JT354_gp01 gene (encoding a hypothetical protein).

### SorTn-seq: transposon mutagenesis

Transposon libraries for SorTnSeq were constructed as previously described (24,25). Briefly, *E. coli* donor strain ST18 harbouring the Tn*5* transposon delivery plasmid pKRCPN2 was conjugated into the recipient strain PCF396 harbouring either the CRISPR3 or CRISPR4 reporter plasmids (pPF1923 or pPF1924). Following conjugation, cells were inoculated into three flasks (2 L) containing 500 ml of LB (supplemented with Km and Cm) at a starting OD_600_ of 0.02 and grown at 30°C with shaking (180 rpm) for 24 h to select for transposon mutants. After outgrowth, cells were pooled (45 ml in total), centrifuged and resuspended in LB to a final OD_600_ of 3 to generate the final Tn library. Aliquots (1 ml) of the library were mixed 1:1 with 50% glycerol and frozen in cryotubes at − 80°C for future use.

### SorTn-seq: fluorescent activated cell sorting (FACS)

Sorting of mutant libraries was performed as previously described (24,25). Briefly, 1 ml of frozen Tn mutant library were subcultured (starting OD_600_ = 0.05) into 30 ml of LB with Cm for reporter plasmid selection, Km for transposon selection and IPTG for mCherry induction. Cells were grown for 16 h to allow expression of the CRISPR-eYFP reporter. Cells were then diluted in 15 ml of phosphate-buffered saline (PBS, 1:30) and sorted using a FACSAria Fusion (BD Biosciences). Despite minimal spectral overlap between eYFP and mCherry, single stain controls (mCherry only-PCF396 + pPF1438; eYFP only-PCF396 + pPF1307; and unstained cells-PCF396) were used to establish a compensation matrix in the BD FACSDiva software (v.8, BD Biosciences). Cells were gated on forward scatter (FSC) and side scatter (SSC) parameters (area, height, width) as seen in (24,25). The mCherry^+^ cells were selected and sorted into three bins based on eYFP fluorescence levels. Gates high and low were each set at approximately 5% of the total, whereas the depleted gate was set at 90%. Cells were sorted under ‘purity’ mode, and approximately 2.0 × 10^7^ cells were sorted per experiment. Sorted cells were recovered in 0.2 ml of PBS. Outgrowth and sorting were performed in triplicate on different days, with each experiment yielding one low, one high, and one depleted fraction (total *n* = 9).

### SorTn-seq: DNA extraction, library preparation and sequencing

Preparation of sorted cells for deep-sequencing was performed as previously described (24,25). Sorted cell fractions (*n* = 9) were centrifuged and DNA was extracted using the DNeasy Blood and Tissue Kit (QIAGEN) following the manufacturer's instructions. Sequencing libraries were constructed using the NEBNext Ultra II FS DNA Library Prep Kit for Illumina. The protocol was modified to have two rounds of PCR enrichment. Round one used customized PCR enrichment primers: PF3140, which binds the NEB adaptor, and PF3139, a biotinylated primer that binds within the Tn. Biotinylated products were captured using Dynabeads M-270 Streptavidin (Invitrogen) following the manufacturer's instructions, and beads were used as the template in the second-round PCR with a nested Tn primer PF3270 and an indexing primer (NEBNext Multiplex Oligos for Illumina). Library quality was assessed on an Agilent Bioanalyzer 2100 using a High Sensitivity DNA Kit. Libraries were further assessed through quantitative PCR (KAPA Library Quantification Kit, Universal, catalogue no. KK4824) using primers PF3124/PF3125 to determine molarity of fragments with Illumina P5/P7 ends (sequences required for flow-cell hybridization). Tn sequencing primer PF2926, along with PF3125, were used to determine the percentage of fragments containing true Tn sequences. Libraries were quantified using a Qubit fluorimeter and dsDNA HS Kit (Thermo Fisher Scientific) and diluted to 10 nM based on Qubit concentration and average fragment size. Libraries were then pooled with 10% PhiX control library and loaded at 1.5 pM (for low diversity) using a MiSeq Reagent Kit v.3 150 cycle kit for Illumina. Libraries were sequenced for 75 cycles (single ended) using custom sequencing primer PF2926 and Illumina Read 1 primer PF3441 (for PhiX library) at the Otago Genomics Facility (OGF). Sequencing with PF2926 generates a 12-nt transposon ‘tag’ to verify reads originating from Tn junctions.

### SorTn-seq: data analysis

SorTn-seq data was analyzed as previously described (24). Samples were de-multiplexed based on index sequence by OGF using standard Illumina software. FASTQ files were trimmed from the 3′-end to 50 nt using trimmomatic (30) and mapped to reference sequences using the SorTn-seq pipeline (31). The following parameters were used to run the bacteria_tradis script: ‘-smalt -smalt_k 10 -smalt_s 1 -smalt_y 0.92 -mm 2 -v -f filelist.txt -t TATAAGAGACAG -r laca.fasta’, where -smalt_ commands specify mapping parameters, -t specifies the transposon tag sequence, -r specifies the reference sequence, -f indicates the files to be processed and -mm indicates the number of mismatches allowed in the Tn tag. Plot files (.insert_site_plot), which tabulate the number of reads at each nucleotide position in the reference sequence (plus and minus strands), were generated by the bacteria_tradis script (32). Subsequent analysis was performed in R using SorTn-seq R scripts (31). Plot files were used to generate a table of unique Tn insertion sites (for each sample). Feature enrichment was determined using differential expression analysis (exact methods—classic) of unique insertions in edgeR (33). The depleted samples served as the control group, against which low and high samples were compared. We considered features with an adjusted *P* < 0.05 (Benjamini–Hochberg false discovery rate (FDR) correction) and a log_2_(fold-change (FC)) > 0.5 as potential regulators.

### Flow cytometry expression analysis of *cas10*, CRISPR3 and CRISPR4 promoters

Flow cytometric quantification of expression from the *cas10*, CRISPR3 and CRISPR4 promoters from the eYFP reporter plasmids (pPF1307, pPF1923 and pPF1924 respectively) was analysed in different *Serratia* strains as described previously (25).

### Conjugation interference assays

To assess the ability of CRISPR-Cas systems to interfere with conjugation, plasmids harbouring protospacer sequences corresponding to the first spacer in either the CRISPR1 (type I-E: pPF724), CRISPR2 (type I-F: pPF722), CRISPR3 (type III-A: pPF1043/pPF2841), CRISPR4 (type III-A: pPF3932) arrays, or control plasmids (pPF719, pPF1043, pPF1621) were conjugated into *Serratia* from *E. coli* ST18 donor cells. The efficiency of plasmid conjugation was calculated as the ratio of transconjugants per total recipients as described previously (34,35).

### Type I-F CRISPR adaptation assays

To assess the effect of *pigU* expression on CRISPR adaptation, a plasmid that induces I-F priming (pPF3807 - harbouring a sequence complementary to spacer 2 from the native I-F CRISPR2 array with an AGA PAM) or control plasmid (pPF3805) were conjugated into *Serratia* WT + control expression vector (pQE-80LoriT) and WT + PigU expression vector (pPF1983) from *E.coli* ST18 donors. Transconjugants were grown in LB with Cm (for priming plasmid maintenance), Ap (for expression plasmid maintenance), IPTG (for PigU induction) and arabinose for mCherry induction. After 24 h, strains were subcultured (1/500) in fresh LB with only Ap (for expression plasmid maintenance) and arabinose (for mCherry induction). Passaging was performed for four days, and each day the density of cultures was measured (OD_600_) and dilutions of cells were frozen for array expansion screening. Aliquots of cultures from each day were also mixed 1:1 with 50% glycerol and frozen at -80°C for future use. CRISPR2 array expansion (indicative of adaptation) was assessed on day two of passaging via PCR (20 cycles) with primers PF1888/PF1990. PCR samples were run at 180 volts on a 2% (w/v) agarose gel with ethidium bromide in sodium borate buffer.

### Phage efficiency of plating (EOP) assay

Strains with chromosomal anti-phage spacers for type I-F (PCF525) and III-A (PCF925) with either control (pQE-80LoriT) or *pigU* overexpression (pPF1983) plasmids were grown overnight. These cultures (100 μl) were used to seed 4 ml of LB top agar (0.35%) containing Ap for plasmids maintenance. The seeded top agar was spread on LB plates to create a bacterial lawn. Phage tenfold dilutions (5 μl) were spotted on these top agar overlays. After incubation of the plates overnight the plaque forming units (PFU) were counted. For plates with no individual plaques (e.g. only lawn clearing or lysis from without visible), the number of plaques was recorded as one in the following dilution without clearing. EOP was calculated by dividing the PFU with the PFU of the control strain (non-targeting, empty vector).

### RNA sequencing and analysis

Overnight cultures of *Serratia* LacA (WT) and HSPIG43 strains were subcultured into 25 ml LB medium in 250 ml flasks (biological triplicates) to a starting OD_600_ of 0.05. Next, the cultures were grown for 12 h at 30°C with shaking at 200 rpm, and 2 ml samples were collected for RNA extraction. The 12 h time point (early stationary phase) was previously established as a point of elevated CRISPR-Cas activity due to the rising density of bacterial populations (22). Bacteria were harvested by centrifugation and the resulting cell pellets were resuspended in 1 ml RNAlater (ThermoFisher Scientific) and stored at −20°C until further processing. Next, the Qiagen RNeasy kit was used to extract total cellular RNA. In addition, the cells resuspended in RLT buffer (Qiagen RNeasy kit) and β-mercaptoethanol were subjected to a bead-beating step of 30 s to ensure full cell lysis. Two μl of TurboDNase (ThermoFisher Scientific) was subsequently added to the samples for 30 min to degrade all the residual genomic DNA (gDNA) and ensure the purity of RNA samples.

The samples were confirmed to be gDNA-free by means of PCR analysis with primers PF796 and PF797 that are designed to amplify the *flhDC* operon of *Serratia*. Further quality control of the resulting RNA samples were performed using the Nanodrop (ThermoFisher Nanodrop one) and 2100 Bioanalyzer (Agilent Genomics) using the High Sensitivity RNA chip. RNA samples were sequenced at Vertis Biotechnologie in Freising, Germany. Ribosomal RNA (rRNA) was depleted using a RiboZero kit (Illumina), and the remaining RNA species were fragmented by means of an ultrasound. Synthesis of antisense cDNA was initiated through ligation of a TruSeq adaptor sequence (Illumina) to the 3′ OH end of the fragmented RNA. Next, the antisense cDNA was purified, followed by a ligation of a 5′ sequencing adaptor to the 3′ end of the antisense cDNA. The cDNA was then amplified using PCR (the number of PCR cycles was dependent on the amount of starting product) and the resulting products were gel fractionated to satisfy the size requirements for Illumina sequencing. Lastly, cDNA libraries were sequenced with the Illumina NextSeq 500 System to an average depth of approximately 10 million reads per library, generating an output in the form of 75 bp demultiplexed reads in FASTQ format.

Generated reads in FASTQ format were initially processed by removing adaptors and low-quality reads using Trimmomatic (30). Additionally, quality assessment of the reads was carried out using FASTQC (36). Bowtie2 (37) was used with default parameters for mapping reads to the reference genome of *Serratia* sp. ATCC 39006 (accession number: CP025085.1), followed by a conversion to BAM format for analysis using SAMtools (38). Statistical analysis was performed using the DESeq2 package in an R environment to identify differentially expressed transcripts with a false discovery rate (FDR) of less than 5% (39).

### Prophage expression analysis

*Serratia* prophages (SP1, SP2, SP3) were previously predicted (26) using PHASTER (40). In this study, we further refined the prophage boundaries through manual curation based on the location of integrases, core host genes, and RNA-seq data.

## Results

### SorTn-seq identifies regulators of type III CRISPR expression

**Figure 1.**
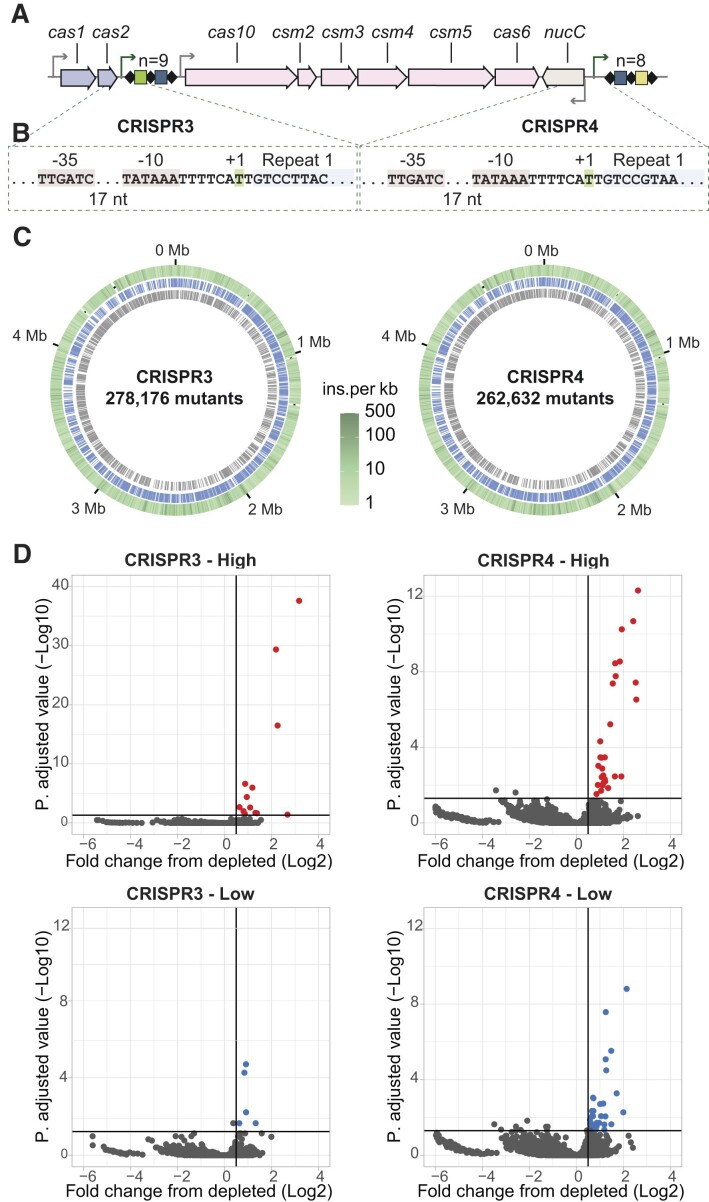
SorTn-seq reveals mutations in genes that significantly impact the *Serratia* CRISPR3 and CRISPR4 promoters. (**A**) Schematic of the type III-A CRISPR-Cas locus in *Serratia*. Interference genes are indicated in pink, adaptation genes in mauve and promoters with black arrows. The CRISPR arrays are depicted with repeats (diamonds) and spacers (coloured rectangles). CRISPR3 and CRISPR4 contain nine and eight spacers, respectively. (**B**) Promoter elements (−35 and −10) and transcriptional start sites (+1) for both CRISPR3 and CRISPR4. Details of the reporter vectors used in the SorTn-seq experiments are shown in Figure S1a–d. (**C**) The *Serratia* genome displaying transposon insertion density for both CRISPR3 and CRISPR4 experiments (outer ring) and genes on the forward (blue) and reverse (grey) strands. Numbers of unique insertions per replicate and in the *high*, *low* and *depleted* samples is provided in Figure S1e. (kb = kilobase, Mb = megabase, ins. = insertions). (**D**) Significant enrichment of mutants in the *low* and *high* samples compared with the *depleted* controls, as determined via exact test for differential expression using *edgeR*. Horizontal lines indicate a *P* value of 0.05 (adjusted for multiple comparisons using Benjamini and Hochberg false discovery rate correction); vertical lines indicate a log_2_ fold change of 0.5. Complete data sets of significantly enriched genes in the CRISPR3 and CRISPR4 SorTn-seq screens are provided in Supplementary Tables S5 and S6, respectively.

*Serratia* has a type III-A CRISPR-Cas system that contains two independent CRISPR arrays (22) (Figure 1A). To identify regulators affecting CRISPR transcription, SorTn-seq experiments were performed. P_CRISPR_-*eYFP* reporters were constructed for the promoter regions of both CRISPR3 and CRISPR4 (Figure 1B and Supplementary Figures S1a–S1d). High density mini-Tn*5* transposon libraries were made using *Serratia* strains carrying either of the reporters. Fluorescence activated cell sorting (FACS) was then used to sort individual transposon mutants based on the level of eYFP fluorescence into ‘*low’*, ‘*high’* and ‘*depleted’* bins. High throughput, Tn*5* insertion site deep-sequencing was then performed on each bin, and the Tn*5* insertion locations were mapped to the *Serratia* genome (Figure 1c and Supplementary Figure S1e). The CRISPR3 and CRISPR4 libraries had approximately 278 000 and 262 000 mutations respectively, corresponding to an insertion on average every ∼18 and ∼19 nt (Supplementary Table S4). Approximately 80 000 unique insertions were obtained across the triplicates for the *depleted* samples for both CRISPR3 and 4 (Supplementary Figure S1e and Supplementary Table S4). This is comparable to what has previously been obtained for the type III-A *cas* operon in *Serratia* (25). Transposon insertions were identified in the *low* and *high* bins for both CRISPR3 and CRISPR4, with a range of ∼14 500–26 000 unique mutations (Supplementary Figure S1f and Supplementary Table S4).

**Figure 2.**
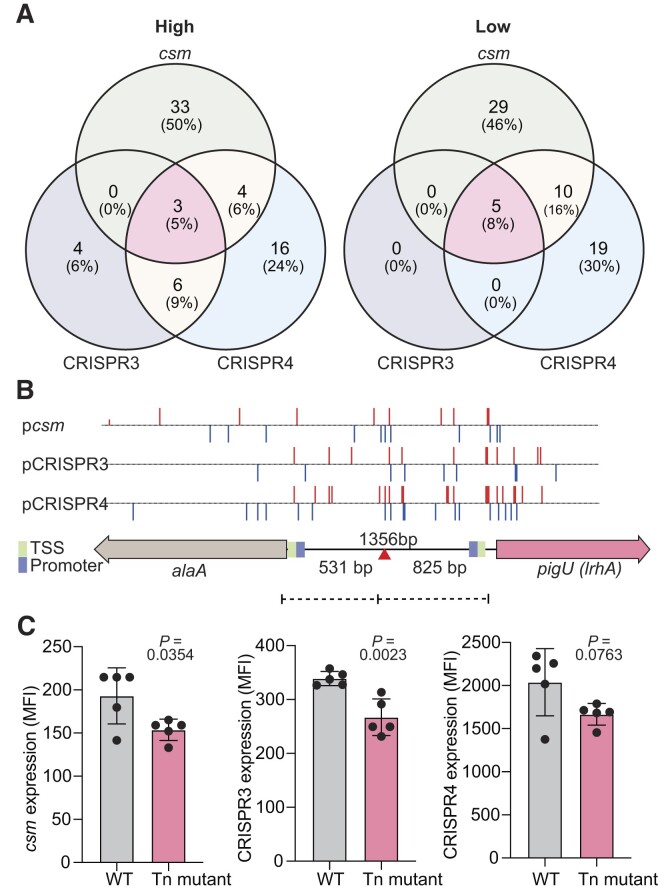
Intergenic insertions between *alaA* and *pigU* (*lrhA*) decrease both *cas* operon and CRISPR array expression. (**A**) Comparison of number of genes and percent (in parentheses) that were significantly enriched across the three different SorTn-seq experiments for the *csm* (25), CRISPR3 and CRISPR4 promoters. Full comparison data is provided in Supplementary Table S7. (**B**) Locations of Tn*5* insertions in the forward (red) or reverse (blue) direction enriched in the *low* bins for the *csm*, CRISPR3 and CRISPR4 promoters relative to the genomic region containing the *alaA* and *pigU* (*lrhA*) genes. Transcriptional start sites (TSS; green) and promoters (blue) are indicated as well as the transposon insertion site in the isolated mutant (HSPIG43; red triangle) (29). (**C**) Mutations in the *alaA-pigU* intergenic region led to decreased CRISPR-Cas expression. Fluorescence of eYFP was assessed for the *csm* (pPF1334), CRISPR3 (pPF1923) and CRISPR4 (pPF1924) promoters in either the WT (PCF396) or the *alaA-pigU* intergenic transposon mutant (Tn mutant, PCF632). Fluorescence normalized by removing empty vector control (pPF1439/pPF1567). Median Fluorescence Intensity = MFI, arbitrary units. Bars are the means and error bars ± SD and individual biological replicates are shown (*n* = 5). Statistical significance was assessed using a two-tailed Student's *t*-test.

Loci of interest were features significantly enriched in the *high* or *low* bins, relative to their respective *depleted* samples (24,25). Using this approach, 13 and five features were identified in the *high* and *low* pools for CRISPR3, respectively (Figure 1D and Supplementary Table S5). For CRISPR4, 29 and 34 hits were identified in the *high* and *low* pools respectively (Figure 1D and Supplementary Table S6). When the CRISPR3 and CRISPR4 *high* pools were compared, many hits were in both, with CRISPR3 having a smaller subset (Figure 2A and Supplementary Table S7). Some genes were excluded from further analysis as their effects were ambiguous, being identified as significantly enriched in both *high* and *low* pools (flagella genes, and two transcriptional regulators (RefSeq locus tags: RS20795/*pigP* (29) and RS17770). It is possible that some of these might have genuine effects, but that the precise positioning of Tn insertions has led to inverse effects (e.g. via disruption or upregulation of a gene). Many genes enriched in these CRISPR promoter screens had predicted roles in outer membrane synthesis (both the outer leaflet lipopolysaccharide (LPS) layer and phospholipid inner leaflet) and peptidoglycan synthesis.

Multiple transposon insertions were clustered within operons involved in LPS synthesis. For example, in two convergent *rfa* operons of three (RS01815-01825) and four (RS01830-01850) genes, two genes in each operon were significantly enriched in the CRISPR screens (Supplementary Figure S2a). These include an O-antigen ligase (RS01825/*rfaL*), an LPS heptosyltransferase III (RS01835/*rfaQ*) and two family 4 glycosyltransferases (GTases) (RS01815/*rfaD* and RS01830). Three of four genes in the RS06430-06445 operon were enriched, including a GTase (RS06430), a polysaccharide biosynthesis protein (RS06435) and a family 4 GTase (RS06445) (Supplementary Figure S2b). Two of three genes were enriched in the RS17850-17860 operon; a nucleotide sugar dehydratase (RS17855) and a NAD-dependent epimerase (RS17860). In addition, both CRISPR screens identified a UTP-glucose-1-phosphate uridylyltransferase (RS13960/*galU*), whereas two hypothetical genes (RS21600 and RS21640) and a specificity factor for O-antigen export (RS21650/*wzt*) within LPS biosynthesis and transport operons, were enriched in the CRISPR4 screen (Supplementary Figure S2c). Therefore, mutations in genes involved in LPS biosynthesis and export result in increased CRISPR array expression.

Other mutations had predicted roles in phospholipid and peptidoglycan synthesis. For example, both screens identified an inner membrane protein that delivers cardiolipin to the outer membrane to increase hydrophobicity in response to outer membrane stress (RS16360/*pbgA*) (41). Likewise, mutations in *asmA* (RS17875), which transfers phospholipids from the inner to outer membrane, were uncovered in both CRISPR promoter screens (Supplementary Figure S2d) (42). The CRISPR4 screen also revealed glutamate racemase (RS03635/*murI*) that generates D-glutamate for peptidoglycan and glycerol-3-phosphate dehydrogenase (RS00755/*glpD*), which is involved in central metabolism and phospholipid synthesis.

Interestingly, in the CRISPR4 *low* pool, mutations in the Rcs stress response pathway were identified (RS00620/*igaA* and RS09790/*rcsA*), supporting our previous work with type III-A *cas* operon regulation (25). This pathway responds to periplasmic and outer membrane stress, so it may be involved in sensing perturbations caused by the other mutations described above. In addition, genes within the Rsm/Csr pathway (e.g. *pigQ*) – and others which influence it (e.g. *crp*, *ptsG* (PTS IIC)) – were identified and previously shown to control type III-A CRISPR-Cas (25,43). Full lists of enriched genes are provided in Supplementary Table S5 and S6. In summary, expression of both type III-A CRISPR arrays responds to changes in outer membrane and peptidoglycan synthesis or composition, and multiple known stress response / signalling pathways also regulate array expression.

### Insertions upstream of PigU decrease type III CRISPR and *cas* expression

We wanted to identify genes that were co-ordinately influencing both CRISPR arrays and the *cas* (*csm*) operon of the type III-A system. We exploited the power of SorTn-Seq to compare our CRISPR3, CRISPR4 results with our previous *csm* dataset (25). This analysis allowed the identification of regulators that were unique or common between the three different promoters (Figure 2A and Supplementary Table S7). A general trend of co-ordinate regulation was observed between CRISPR3 and CRISPR4 as discussed above. Five and eight % of all significant loci were shared between all three promoters (*csm*, CRISPR3 and CRISPR4) in the *high* and *low* pools, respectively (Figure 2A). We focused on loci that affected the expression of all three promoters. As mentioned above, ambiguous hits (in both *high* and *low* pools) were excluded. Mutations in a DNA helicase (*dinG*) and in a *pigU-alaA* intergenic region were shared between these three SorTn-seq experiments. We decided to focus on the intergenic region between the *pigU* and *alaA* genes, since it was enriched for transposon insertions in the *low* pools for all three promoters (Figure 2B) and its role in CRISPR-Cas expression was unexplored. AlaA is a glutamate-pyruvate aminotransferase involved in alanine biosynthesis (44) and PigU is a DNA-binding LysR-type transcriptional regulator (LTTR), that is homologous to *Pectobacterium carotovorum* HexA (45), *Dickeya dadantii* PecT (46) and *E. coli* LrhA (47) (Supplementary Figure S3). These LTTRs have roles in regulating flagella-based motility, secondary metabolites and plant cell-wall degrading enzymes (45,46,48).

Previously, a random transposon mutagenesis had identified an insertion in this same *alaA-pigU* intergenic region that resulted in decreased red pigment (prodigiosin) and carbapenem antibiotic production in *Serratia* (29). The position of this transposon insertion was similar to insertions enriched in our SorTn-seq experiments for the different CRISPR-Cas promoters (Figure 2B; *compare red triangle with red and blue lines*). Therefore, to test the effect of mutation in this region on CRISPR-Cas expression, we made a double mutant of this transposon mutant with the pigmentless (Δ*pigA-O*) strain of *Serratia* used for fluorescence assays. The eYFP reporter plasmids for the *cas* operon and CRISPR3 and CRISPR4 were introduced into this strain and expression was assessed by flow cytometry. Expression of the *csm* promoter (*cas* operon) and the CRISPR3 and CRISPR4 promoters was lower in the transposon mutant than in the isogenic control (Figure 2C). Therefore, insertions in the intergenic region between *alaA* and *pigU* decrease type III-A CRISPR-Cas expression.

### Overproduction of PigU represses type III CRISPR-Cas interference

**Figure 3.**
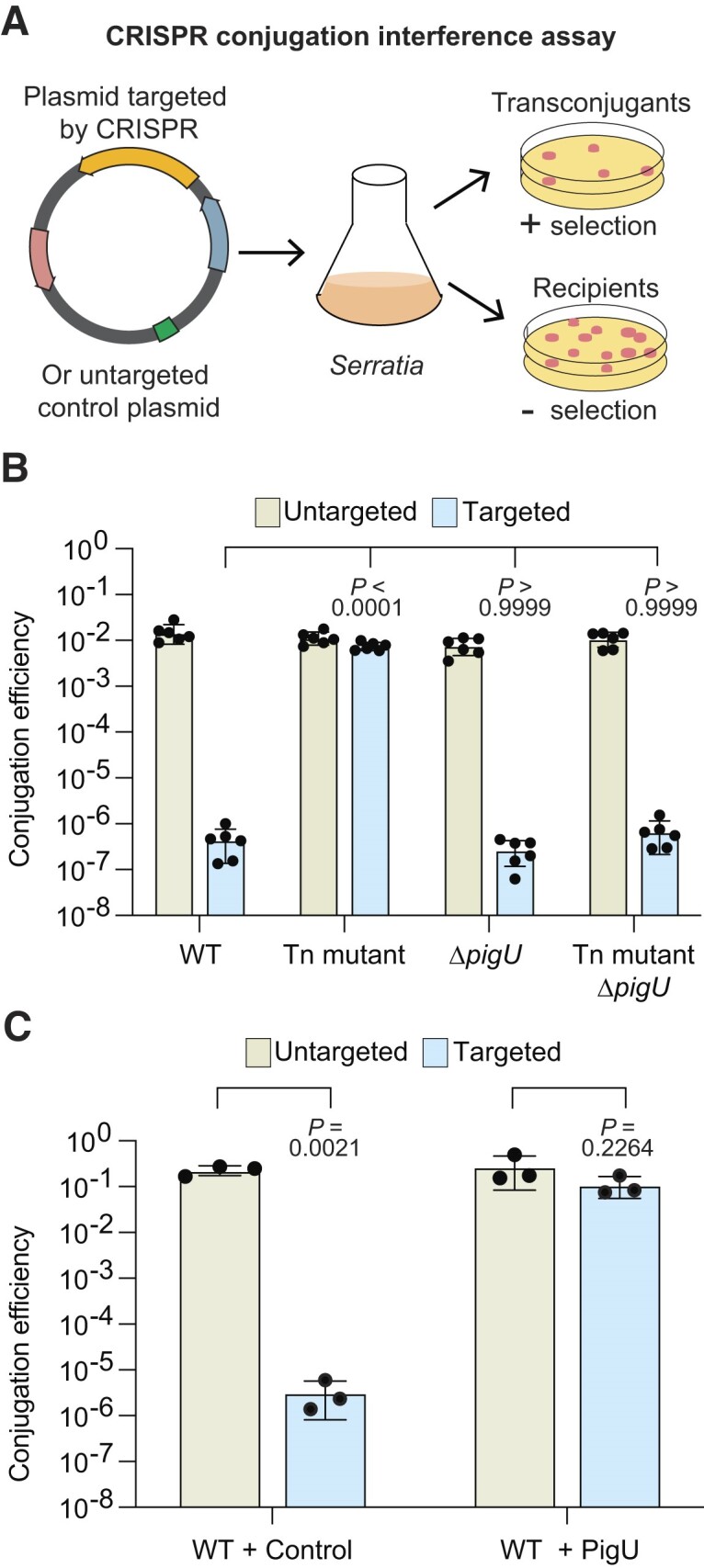
PigU represses type III-A CRISPR-Cas interference. (**A**) Schematic of the conjugation efficiency CRISPR-Cas interference assays. Plasmids with (targeted) or without (untargeted) a protospacer matching spacer 1 in the particular CRISPR array are conjugated into *Serratia* strains containing the native CRISPR-Cas systems. Interference is assessed by measuring conjugation efficiency by plating with and without selection for the transconjugants and recipients. (**B**) CRISPR conjugation interference assay of an untargeted plasmid (pPF781) or a plasmid targeted (pPF1043) by CRISPR3 spacer 1 in the type III-A system in WT (PCF396), an *alaA-pigU*intergenic transposon mutant (Tn mutant, PCF632), a Δ*pigU* mutant (Δ*pigU*, PCF708) and an *alaA-pigU* intergenic, Δ*pigU* double mutant (Tn mutant Δ*pigU*, PCF709). Statistical significance of conjugation efficiency (targeted strains) assessed using a one-way ANOVA (*P*< 0.0001) with Dunnett's multiple comparisons test against the WT (PCF396). (**C**) CRISPR conjugation interference assay as in (B) in WT (LacA) either containing a vector control (pQE-80LoriT; Control) or a plasmid expressing PigU (pPF1983; PigU). Statistical significance was assessed using a two-tailed Student's *t*-test of untargeted versus targeted. In (B) and (C), bars are the means and error bars ± SD. Individual biological replicates are shown for (B) (*n*= 6) and (c) (*n*= 3).

Since the *alaA-pigU* intergenic transposon insertions led to decreased expression of the *csm*, CRISPR3 and CRISPR4 promoters, we predicted that type III-A CRISPR-Cas interference would be lower. Type III-A CRISPR-Cas systems function through sequence-specific RNA targeting that triggers a series of enzymatic activities involved in invader defence (49). Cas6 generates crRNAs and the Cas protein complex assembles on these guides. The complex recognises and binds complementary RNAs, such as those produced by invader gene expression. RNA recognition triggers the HD domain in Cas10 to cleave ssDNA (e.g. within the transcription bubble) and the Cas10 palm domain catalyses cyclic oligoadenylate (cOAs) formation. These cOA molecules are bound by accessory proteins that are often sequence non-specific RNases and DNases that cleave phage and host nucleic acids (49). In *Serratia*, the accessory nuclease is NucC (Figure 1A), which responds to cA_3_ and cleaves dsDNA (50–52). The *Serratia* type III system provides protection against plasmids and phages (22,25,43,50,51). To test the effect of the *alaA-pigU* intergenic mutation on type III-A activity, we used a plasmid conjugation-based CRISPR-Cas interference assay using an untargeted control plasmid, or a plasmid with sequences expressed from an arabinose inducible promoter to generate RNA targets of spacer 1 of CRISPR3 (Figure 3A) (22). The untargeted plasmid was acquired efficiently by conjugation in the WT and intergenic Tn mutant, whereas the targeted plasmid was inhibited >10 000-fold in the WT background (Figure 3B). In contrast, type III-A interference was completely abolished by the *alaA-pigU* intergenic transposon mutation (Figure 3B). Likewise, protection provided by spacer 1 of the CRISPR4 array was similarly reduced in the *alaA-pigU* intergenic transposon mutant background (Supplementary Figure S4a). These results highlight the power of these SorTn-seq screens to identify regulators of physiological relevance.

Despite the clear impact of this mutation on type III-A CRISPR-Cas activity, whether this was related to AlaA or PigU (or other effects) was unknown. Interestingly, in *E. coli*, insertion of a transposon in the *alaA-lrhA* intergenic region resulted in overexpression of *lrhA* (47). Since PigU homologues regulate diverse functions in different proteobacteria, we hypothesised that the intergenic mutations also led to increased PigU in *Serratia*, which in turn repressed CRISPR-Cas. In agreement with this model, deletion of *pigU* in the intergenic transposon mutant background restored interference to this double mutant (Figure 3B). This suggested that *pigU* expression is normally low in these growth conditions and that the intergenic mutation has led to a PigU increase, which is acting as a repressor. Indeed, a single mutation (deletion) of *pigU* had no effect on interference levels (Figure 3B), presumably due to its low basal expression in these growth conditions. To confirm that PigU overexpression was responsible for decreased CRISPR-Cas activity, recipient cells containing a plasmid expressing *pigU* were tested in the conjugation efficiency assay. In agreement, PigU overexpression in the WT background abolished type III-A interference (Figure 3C) and mimicked the phenotype of the *alaA-pigU* intergenic mutant. Interestingly, this effect did not require any inducer (IPTG) and demonstrated that leaky expression of PigU was sufficient for these strong phenotypic effects. In summary, mutation of the intergenic region leads to increased PigU, which represses type III CRISPR-Cas interference.

### PigU represses type I-F CRISPR-Cas plasmid interference and adaptation

**Figure 4.**
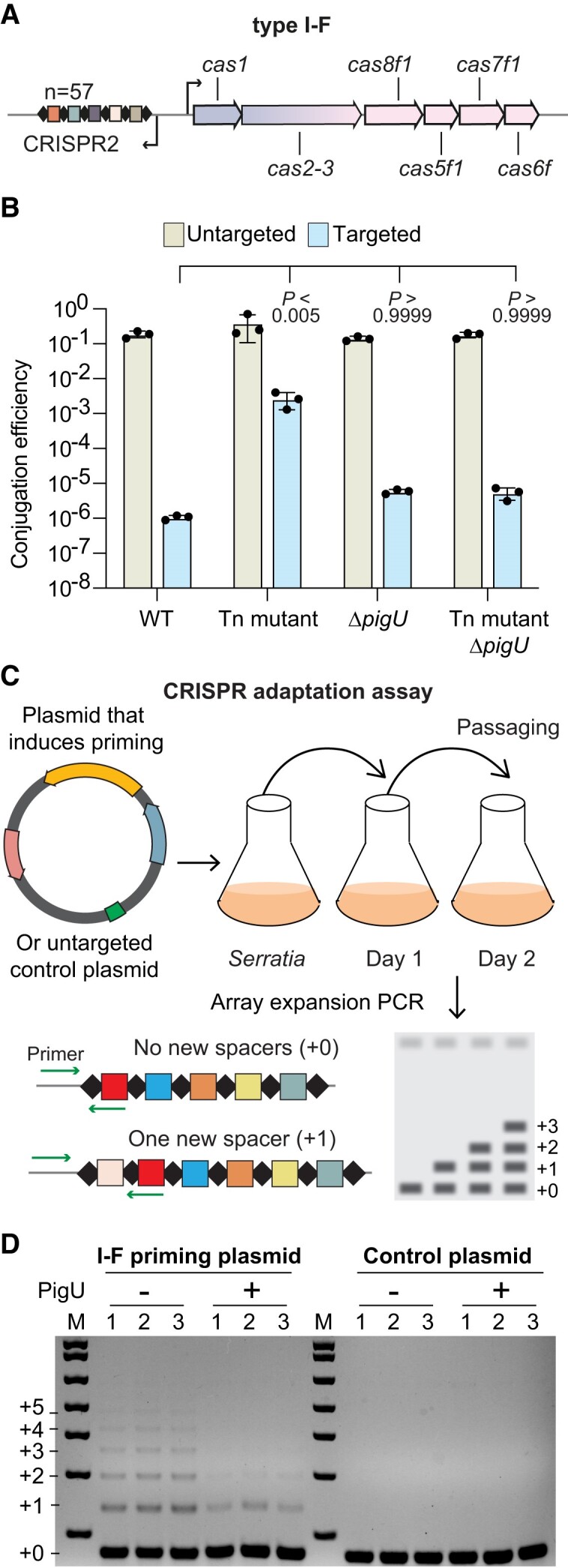
PigU represses type I-F CRISPR-Cas interference and adaptation. (**A**) Schematic of the type I-F CRISPR-Cas locus in *Serratia*. Interference genes are indicated in pink, adaptation genes in mauve and promoters with black arrows. The CRISPR2 array contains 57 spacers and is depicted with repeats (diamonds) and spacers (coloured rectangles). (**B**) Conjugation efficiency of an untargeted plasmid (pPF719) or a plasmid targeted (pPF722) by the type I-F system in WT (LacA), an *alaA-pigU* intergenic transposon mutant (Tn mutant, HSPIG43), a Δ*pigU* mutant (Δ*pigU*, PCF720) and an *alaA-pigU* intergenic, Δ*pigU* double mutant (Tn mutant Δ*pigU*, PCF707). Bars are the means and error bars ± SD. Individual biological replicates are shown (*n*= 3). Statistical significance of conjugation efficiency (targeted strains) was assessed using a one-way ANOVA (*P*= 0.0029) with Dunnett's multiple comparisons test against the WT (LacA). (**C**) Schematic of the CRISPR adaptation assay. Cells harbouring either a vector inducing priming or control vector are passaged for multiple days, and array expansion is assessed by PCR. (**D**) Population level assessment of adaptation in the CRISPR2 array at day two. Array expansion was measured in WT (LacA) cells harbouring a plasmid that induces I-F priming (I-F priming plasmid; pPF3807) or a non-targeted control (control plasmid; pPF3805), in the presence (+ pPF1983) or absence (− pQE-80LoriT) of PigU overexpression. Individual biological replicates are shown (*n*= 3). (M = molecular weight marker).

We previously identified type III-A CRISPR-Cas system regulators that also controlled the type I CRISPR-Cas systems in *Serratia* (22,25,43). We hypothesised that PigU may also regulate the DNA-targeting type I CRISPR-Cas systems in *Serratia*. To test this, we performed conjugation-based CRISPR interference assays using either an untargeted control plasmid, or plasmids with sequences targeted by spacer 1 in CRISPR1 (type I-E) or CRISPR2 (type I-F) (Figure 4A and Supplementary Figure S4b). In both type I systems, untargeted plasmids were acquired efficiently by conjugation in all strains and targeted plasmids were strongly inhibited in the WT background (Figure 4B and Supplementary Figure S4c). Whereas there was no effect on type I-E interference in the strain with elevated PigU (Supplementary Figure S4c), type I-F interference was severely attenuated (>1000-fold; Figure 4B). In agreement with the requirement of *pigU* for repression of type III-A interference, deletion of *pigU* in the transposon mutant restored targeting of the type I-F CRISPR-Cas system to WT levels (Figure 4B).

Since PigU repressed type I interference, we hypothesised that CRISPR adaptation would also be inhibited. To measure CRISPR adaptation, a priming assay was performed using a plasmid that increases new type I-F spacer acquisition, due to a matching spacer but an imperfect protospacer adjacent motif (PAM). Adaptation assays were performed with or without PigU expression from a plasmid in a WT background, and new spacer acquisition was visualised as CRISPR array expansion in the bacterial population by using PCR (Figure 4C). No adaptation was detected in the un-primed plasmid control, whereas type I-F CRISPR primed adaptation was reduced by PigU expression (Figure 4D). In conclusion, in addition to its effects on type III-A activity, PigU also represses type I-F CRISPR-Cas interference and adaptation.

### PigU controls type I and III-mediated phage resistance

**Figure 5.**
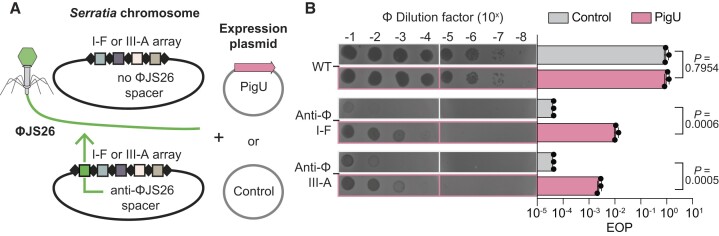
PigU decreases phage immunity of both the type I-F and III-A CRISPR-Cas systems. (**A**) Schematic of the experimental setup. Strains of *Serratia* with either a control (pQE-80LoriT) or PigU expression (pPF1983) plasmid contained no additional spacer (LacA) or an additional single spacer targeting phage JS26 at the leader-end of the CRISPR arrays for type I-F (CRISPR2; PCF525) or type III-A (CRISPR3; PCF925) systems. These strains were infected with phage JS26 and plaquing assessed. (**B**) Representative spot titre results of phage JS26 on the WT control strain and strains with a type I-F or III-A spacer targeting the phage in the presence or absence of PigU expression (*left*). Quantification of the efficiency of plating (EOP) relative to the WT with the control vector. Bars are the means and error bars ± SD and individual biological replicates are shown (*n*= 3). Statistical significance was assessed using two-tailed Student's *t*-tests of strains carrying control plasmid (pQE-80LoriT) versus the PigU expression plasmid (pPF1983).

Since PigU repressed both type I and III CRISPR-Cas interference against plasmid uptake by conjugation, we wanted to test if it also affected the phage resistance response. We used strains of *Serratia* that contained a single spacer that targeted phage JS26 in either the type I-F (CRISPR2) or type III-A (CRISPR3) chromosomal arrays (Figure 5A). A control vector or a PigU expression plasmid in the WT and these JS26-targeting strains were infected with phage JS26 and their plaquing examined quantitatively (Figure 5B). JS26 is a siphovirus with a dsDNA genome of ∼64 kb that is sensitive to type I-F and III-A CRISPR-Cas systems in *Serratia* (28,50,53,54). The single chromosomal spacers in either the type I-F or type III-A systems reduced the plaquing of JS26 by almost 10^5^-fold, demonstrating a strong CRISPR-Cas response (Figure 5B). Expression of PigU reduced the strength of CRISPR-Cas immunity by ∼10^3^ and ∼10^2^-fold for the type I-F and III-A systems, respectively. Therefore, PigU represses both type I and III CRISPR-Cas immunity against phages.

### PigU is a global regulator of secondary metabolism, motility and CRISPR-Cas

**Figure 6.**
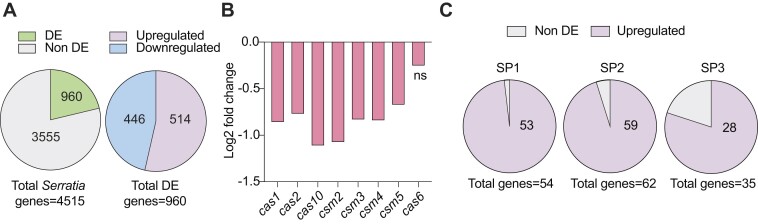
PigU is a global regulator of gene expression in *Serratia*. (**A**) Summary of differentially expressed (DE) genes in the *alaA-pigU* intergenic transposon mutant as compared against the WT control using DESeq2 (Supplementary Table S8). (**B**) Downregulation of the type III-A *cas* genes in the *alaA-pigU* intergenic transposon mutant. Log2 fold change and statistical significance was assessed via DESeq2 (Supplementary Table S8; not significant (ns)). (**C**) Upregulation of three predicted *Serratia* prophages: SP1(RS07685-RS07960; 54 genes), SP2 (RS14105-RS14425; 62 genes) and SP3 (RS14800-RS14975; 35 genes).

To further investigate the role of PigU, RNA sequencing (RNA-seq) was used to identify changes in RNA abundance between a WT and the *alaA-pigU* transposon mutant. Both the WT and *alaA-pigU* mutant showed similar growth profiles and RNA was extracted at early stationary phase (12 h) for sequencing (Supplementary Figure S5a). Using DESeq2 (39), we identified 960 genes (∼21% of the genome) having significant differential expression, with 514 upregulated and 446 downregulated in the transposon mutant compared to the WT (Figure 6A and Supplementary Table S8). First, we examined our hypothesis that the transposon mutation led to increased *pigU* expression. Consistent with our earlier work suggesting that PigU was elevated in the mutant (Figure 3), *pigU* mRNA was ∼1.6-fold higher than the levels in the WT (Supplementary Table S8 and Supplementary Figure S5b). Interestingly, genes immediately upstream (*alaA*) and downstream (RS10445) of *pigU* were also significantly upregulated (Supplementary Table S8), consistent with LTTRs controlling the expression of adjacent genes (55). Interestingly, in the transposon mutant, there were more reads mapping upstream of the transposon insertion site—located 825 bp upstream of *pigU* (Supplementary Figure S5c). While there are no annotated features in this region, the finding that insertions there in both *Serratia* and *E. coli* lead to *pigU/lrhA* overexpression, and that full *lrhA* complementation in *Pantoea stewartii* required ∼921 bp of the intergenic region (56) suggests there could be important regulatory elements, such as sRNAs, in this location. Furthermore, the levels of the type III-A *cas* genes for the interference (*csm*) operon were significantly decreased in the mutant, with the exception of *cas6*, the last in the transcript (Figure 6b). The gene encoding the accessory nuclease NucC, which is expressed convergently with the *cas* operon under a different promoter, was not altered (Supplementary Table S8). We saw a similar lack of *nucC* regulation under the Rsm system previously (43). Expression of both type III-A adaptation genes (*cas1* and *cas2*) was significantly lower in the mutant (Figure 6B). Surprisingly, no significant differences in the type I-F genes were detected (Supplementary Table S8), suggesting that PigU-mediated control of this system involves post-transcriptional regulation.

This *alaA-pigU* intergenic transposon mutant of *Serratia* had previously been shown to result in reduced production of two antimicrobial secondary metabolites: the red tripyrrole pigment prodigiosin and the beta-lactam antibiotic, carbapenem (29). In agreement, RNA-seq showed that expression of several genes in operons encoding these two antimicrobial molecules (prodigiosin; RS20700–RS20630 and carbapenem; RS09910–RS09950) was significantly lower in the transposon mutant (Supplementary Table S8). Since PigU homologues affect motility (45,48), we also examined the regulators (*flhDC*) and structural genes required for flagella biosynthesis. Most genes for flagella-mediated motility and neighbouring chemotaxis genes were increased in the transposon mutant (Supplementary Table S8), consistent with an *lrhA* mutant transcriptome in *E. coli* (48). *Serratia* also floats using gas vesicles encoded by RS01290–RS01190 (57). Gas vesicles give *Serratia* a white opaque phenotype and are inversely controlled with flagella. Consistent with the effects on flagella, the elevated levels of *pigU* in the transposon mutant led to a translucent colony colour (29) and the gas vesicle genes were repressed (Supplementary Table S8). In summary, increased PigU levels led to pleiotropic effects, with decreased transcripts for CRISPR-Cas, secondary metabolism and gas vesicles, whereas expression of flagella genes were increased.

Since PigU controls flagella and mutations in *flhDC* were also identified during SorTn-seq, we investigated whether changes in flagella alter CRISPR-Cas immunity. Previously, we demonstrated that transposon insertion in *flhD or flhC* led to small decreases in *csm* expression levels (25). Interestingly, deletion of either *flhD* or *flhC* did not significantly alter type III-A interference against plasmid conjugation (Supplementary Figure S6a). To determine if flagella are required for PigU-mediated CRISPR-Cas repression, we overexpressed *pigU* and performed a conjugation efficiency assay in the presence or absence of *flhDC*. Conjugation was still severely attenuated upon deletion of *flhDC* in a PigU overexpression strain, indicating that *flhDC* is not required for PigU-mediated CRISPR-Cas (Supplementary Figure S6b). In addition, overexpression of *flhDC* in the Δ*pigU* mutant had no appreciable effect on conjugation, with interference levels similar to that of WT (Supplementary Figure S6c). These results indicate that, while PigU controls expression of *flhDC*, these changes in flagella do not contribute to CRISPR-Cas repression.

### PigU influences prophage, toxin–antitoxin and tRNA gene expression

Surprisingly, some of the most strongly upregulated genes in the transposon mutant were from three prophages—named SP1, SP2 and SP3 (26), with ∼93% of their genes upregulated (Figure 6C and Supplementary Table S8). These prophages are active under certain conditions, as excision and phage particle release has been previously demonstrated in response to *rsmA* deletion in *Serratia* (58). Prophage induction is often associated with the SOS response, and while *recA* expression was upregulated ∼2-fold in the transposon mutant, downstream DNA-repair genes such as *uvrA/B* were not differentially expressed (Supplementary Table S8). Interestingly, overexpression of *rcsA*, which encodes a component of the regulator of capsule synthesis (Rcs) stress response, results in RecA-independent *E. coli* lambdoid prophage induction (59,60). In the transposon mutant, *rcsA* (RS09790) was upregulated ∼3-fold (Supplementary Table S8). Expression of *rcsA* is directly controlled by the PigU homolog LrhA in *P. stewartii* (61). This might suggest that increased PigU in *Serratia* induces prophages via RcsA, in a pathway distinct from the canonical SOS-response.

Components of seven toxin-antitoxin (TA) systems were also differentially expressed in the transposon mutant (Supplementary Tables S8 and S9) and multiple TA systems are known to provide phage defence (62). Previously, we characterised the TA repertoire of *Serratia*, which contains 32 predicted TA systems (26). In almost all cases, increased *pigU* expression led to upregulation of TA systems (Supplementary Table S9). We also observed differential expression of 55 tRNA-related genes (45 tRNAs and 10 tRNA-related genes (e.g. synthases/ligases/methyltransferases). In nearly all cases (51/54 genes), expression was upregulated (Supplementary Table S8). Whether tRNAs may be induced in response to TA system activation or prophage induction (or both) is unknown. In summary, the *alaA-pigU* transposon mutation resulted in increased prophage expression and elevation of TA systems.

### PigU controls expression of sRNAs that regulate *rpoS* translation

Interestingly, upon *pigU* overexpression, we also observed decreased transcripts for *rpoS*, which encodes the stationary-phase sigma factor RpoS (σ^S^) (Supplementary Table S8). RpoS levels are regulated at transcriptional, translational, and post-translational levels, and involves non-coding small RNAs (sRNAs) (63). The sRNAs RprA and ArcZ, assisted by the post-transcriptional chaperone Hfq, bind the *rpoS* 5′ UTR to favour secondary structures that allow translation (63). In *Serratia, pigU* overexpression downregulated *rprA* (Supplementary Figure S7a-S7b, Supplementary Table S8), consistent with *E. coli* (64). A homology search revealed a putative *arcZ* homolog in *Serratia*, which was downregulated by PigU (Supplementary Figure S7c-S7d). While ArcZ controls translation of the PigU homologs PecT and HexA in *Dickeya dadantii* and *Photorhabdus / Xenorhabdus* spp. (65,66), our data suggests that the inverse (PigU regulation of *arcZ)* occurs in *Serratia*. Interestingly, expression of another sRNA, CyaR, which is negatively regulated by ArcZ (67), is upregulated upon *pigU* overexpression in *Serratia* (Supplementary Figure S7e-S7f). CyaR represses *rpoS* translation, and the upregulation upon *pigU* overexpression may be a result of lowered transcription of *arcZ* (Supplementary Figure S7c-S7d). In *E. coli*, it is thought that LrhA-mediated control of *rpoS* translation likely requires another Hfq-dependent sRNA (in addition to RprA (64)), thus ArcZ is a candidate worth investigating.

To test whether *rpoS* influences CRISPR-Cas immunity, we performed type III-A conjugation interference and phage infection assays with an *rpoS* mutant (68). There was a slight (∼5-fold) decrease in type III-A plasmid targeting in the *rpoS* mutant (Supplementary Figure S7g), a more subtle phenotype than that of the *pigU* overexpression mutant (Figure 3B). For phage infection, there was no detectable difference in protection in a strain harbouring a type III-A anti-phage spacer upon mutation of *rpoS* (Supplementary Figure S7h). Interestingly, the *rpoS* mutation affected phage JS26 plaque phenotype (Supplementary Figure S7i). These results suggest that RpoS levels do not substantially contribute to CRISPR-Cas regulation, and the strong repression observed upon *pigU* overexpression is due to an alternative pathway.

### PigU inhibition of CRISPR-Cas is predominantly independent of known CRISPR-Cas regulators

We investigated if increased PigU was acting through other known CRISPR-Cas regulators to influence adaptive immunity in *Serratia* (Supplementary Figure S8a). RsmA (CsrA) is a post-transcriptional regulator which represses CRISPR-Cas (43). Levels of free RsmA are modulated by the sRNA RsmB (CsrB) and PigX (CsrD), which degrades RsmB. Upon *pigU* overexpression, *rsmB* was elevated (Supplementary Figure S8b, c) and *csrD* was decreased (Supplementary Table S8). These conditions would result in greater RsmA sequestration and more Cas translation due to decreased repression of target *cas* gene mRNAs (43). Therefore, the CRISPR-Cas repression mediated by PigU cannot be explained through effects on the Rsm pathway. QS also regulates CRISPR-Cas in *Serratia* (22) and the PigU homolog HexA in *E. carotovora* activates production of the QS signal (69). However, in *Serratia*, PigU did not influence transcript levels from the *smaIR* QS genes (Supplementary Table S8), or affect QS signal levels (29). Finally, both PigU and the Rcs pathway, a previously characterized regulator of CRISPR-Cas in *Serratia* (43), regulate transcription of the small RNA *rprA* (70). In *Serratia, rprA* expression is increased during Rcs activation (43) and decreased upon *pigU* overexpression (Supplementary Figure S7a, Supplementary Table S8). Deletion of *rprA* or *rpoS* (Supplementary Figure S7e) had no (43), and very little (Supplementary Figure S7g), effect on CRISPR-Cas immunity, respectively. In summary, although various CRISPR-Cas regulators are influenced by PigU upregulation, PigU is likely acting through independent pathways to repress adaptive immunity.

## Discussion

Previously, we developed SorTn-seq to systematically identify regulators of bacterial gene expression and used it to identify factors which influence type III-A *cas* (*csm*) expression in *Serratia* (25). Here, we performed SorTn-seq of both type III-A CRISPR arrays (CRISPR3 and CRISPR4) to produce a comprehensive analysis of type III-A CRISPR-Cas regulation in *Serratia*. We discovered several mutations which affect CRISPR expression, including those which likely alter outer membrane/cell surface structures including LPS, O-antigen, and peptidoglycan. Interestingly, many of these genes were also enriched during *cas* (*csm*) SorTn-seq. While the exact mechanism of regulation remains to be elucidated, these findings indicate that changes in outer membrane biogenesis can lead to coordinate changes from both CRISPR and *cas* (*csm*) gene promoters.

Of particular interest were mutations mapping to the intergenic region between *alaA* and *pigU* that were enriched in the low expression bins of all three type III-A SorTn-seq screens. Overexpression of *pigU* suppressed both type III-A and type I-F CRISPR-Cas targeting, reducing protection against both plasmids and phage. PigU repressed adaptation by the type I-F system, thus, all stages of CRISPR-Cas immunity—expression, interference and adaptation—are controlled by PigU. Although type III adaptation has not been observed in *Serratia*, expression of genes encoding the adaptation machinery (type III-A *cas1* and *cas2*) also falls under PigU control—suggesting PigU may also influence type III spacer acquisition. Interestingly, type I-E immunity was not affected, indicating that the three CRISPR-Cas systems in *Serratia* are not always co-ordinately regulated. In agreement with the importance of PigU as a regulator of CRISPR, *E. coli* LrhA was recently shown to activate type I-E CRISPR-Cas immunity in an *hns*/*leuO* double mutant through binding to the *cas* promoter (71).

In addition to its role as a CRISPR-Cas repressor, RNA-seq revealed that PigU is also a pleiotropic regulator, controlling genes involved in secondary metabolism and prophage / TA system induction. Interestingly, six of eight genes in a predicted LPS and O-antigen biosynthesis operon that were enriched in the SorTn-seq (Supplementary Figure S2c) were also differentially expressed in the transposon mutant (Supplementary Table S10), suggesting some shared regulatory pathways. However, the cellular cues which control *pigU* expression remain largely unknown. In *P. stewartii*, the QS master regulator EsaR directly activates *lrhA* expression (72,73). Therefore, similarly, *pigU* expression in *Serratia* may be under QS control, but this currently unknown. In *E. coli*, mutation of *ftsK*, encoding a DNA translocase involved in chromosome segregation and cell division (74) leads to *lrhA* overexpression, which influences levels of the stationary-phage sigma factor RpoS (64,75). It is likely that some of the global transcriptomic shifts observed upon *pigU* overexpression are a result of decreased levels of RpoS. However, in *Serratia*, these changes do not contribute substantially to CRISPR-Cas immunity. Likewise, PigU appears to repress adaptive immunity in *Serratia* independently of known CRISPR-Cas regulators, although significant regulatory interconnection exists (Supplementary Figure S8).

Overall, we have demonstrated the utility of the SorTn-seq method to identify regulators of non-coding RNA (CRISPR array) expression. We have uncovered a major regulator of type III CRISPR-Cas that co-ordinately controls *cas* operon and CRISPR array expression to influence CRISPR-Cas interference and adaptation. Importantly, our study further demonstrates that CRISPR-Cas systems are often ‘wired’ into major conserved regulatory networks that have pleiotropic effects on cellular processes. The type III-A system in *Serratia* is present in an integrative and conjugative element, and is likely the most recent CRISPR-Cas system acquired by this strain. Type III-A regulation by QS (22), Rcs (25), Rsm (43) and now LrhA (PigU), support the idea that following this horizontal gene transfer event, this defence system has connected into the existing regulatory networks that co-ordinate diverse cellular behaviours.

## Supplementary Material

gkad1165_supplemental_files

## Data Availability

Raw reads from SorTn-seq analyses and RNA-seq have been deposited at the NCBI Sequence Read Archive (SRA) under BioProject accession number PRJNA1020058. Processed RNA-seq data have been deposited at the NCBI Gene Expression Omnibus (GEO) under accession number GSE243790. Data analysis pipeline and scripts used for SorTn-seq analyses are available at http://doi.org/10.5281/zenodo.4554398 (31). Reference sequences and annotations used in this study are available through NCBI: *Serratia* sp. ATCC 39006 LacA (CP025085.1 / assembly GCF_002847015.1_ASM284701v1) and phage JS26 (NC_053012.1 / assembly GCF_009662515.1_ASM966251v1).

## Notes

Present address: Hannah G. Hampton, Cawthron Institute, Nelson, New Zealand.

Present address: Mariya S. Yevstigneyeva, Axion BioSystems, Eindhoven, Netherlands.

Present address: Zacharie S.M. Paquet, Belgian Defence Laboratories (DLD), Brussels, Belgium.
