# Supplementary material for: CRISPR-Cas immunity is repressed by the LysR-type transcriptional regulator PigU
Source: Nucleic Acids Res. 2023 Dec 7;52(2):755–68. doi: 10.1093/nar/gkad1165 (PMC10810281; doi:10.1093/nar/gkad1165)
Supplement: gkad1165_supplemental_files [file gkad1165_supplemental_files.zip › Smith_et_al_2023_NAR_supplementary.pdf]

Supplementary Information

CRISPR-Cas immunity is repressed by the LysR-type transcriptional regulator PigU

Leah M. Smith[1,2,5], Hannah G. Hampton[1], Mariya S. Yevstigneyeva[1], Marina Mahler[1,2,5], Zacharie S. M. Paquet[1,3] and Peter C. Fineran[1,2,4,5*]

[1]Department of Microbiology and Immunology, University of Otago, PO Box 56, Dunedin 9054, New Zealand
[2]Genetics Otago, University of Otago, PO Box 56, Dunedin 9054, New Zealand
[3]Laboratory of Microbiology, Department of Agrotechnology and Food Sciences, Wageningen University, Dreijenplein 10, 6703 HB Wageningen, The Netherlands
[4]Bioprotection Aotearoa, University of Otago, PO Box 56, Dunedin 9054, New Zealand
[5]Maurice Wilkins Centre for Molecular Biodiscovery, University of Otago, PO Box 56, Dunedin 9054, New Zealand

Present addresses: HGH; Cawthron Institute, Nelson, New Zealand. MSY; Axion BioSystems, Eindhoven, Netherlands. ZSMP, Belgian Defence Laboratories (DLD), Brussels, Belgium.

[*]For correspondence: peter.fineran@otago.ac.nz

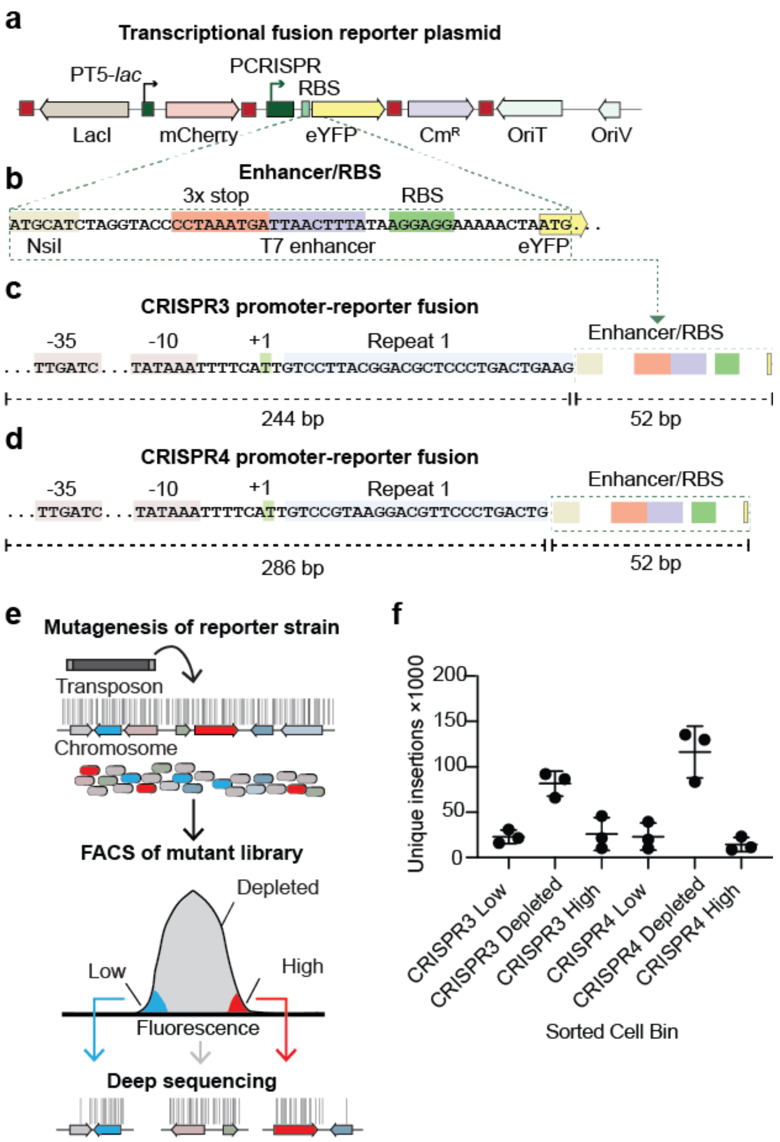

Figure S1. CRISPR reporter plasmids and number of unique transposon insertions following SorTn-seq. a. Overview of the transcriptional reporter plasmid used for the CRISPR promoters for SorTn-seq (pPF1567). mCherry control fluorescence is regulated by a T5/lac promoter to enable induction by IPTG due to de-repression of LacI. The plasmid backbone also contains the *cat* gene for chloramphenicol resistance (Cm^R), an RP4 oriT for mobilisation by conjugation and a ColE1 (pMB1) oriV. CRISPR (or other) promoters of interest can be introduced in front of the eYFP gene. **b.** Detailed schematic of the 3x STOP, T7 enhancer and RBS region (based on the translation initiation region from Miller and Lindow (1)) in front of eYFP to enable efficient translation from any transcripts generated from the **c.** CRISPR3 (pPF1923) or **d.** CRISPR4 (pPF1924) promoters. **e.** Schematic of SorTn-seq workflow. **f.** Number of unique transposon insertions in the three different sorted bins (*low*, *depleted* and *high*) for both the CRISPR3 and CRISPR4 SorTn-seq experiments. Results from the individual replicate SorTn-seq experiments are shown (*n*=3).

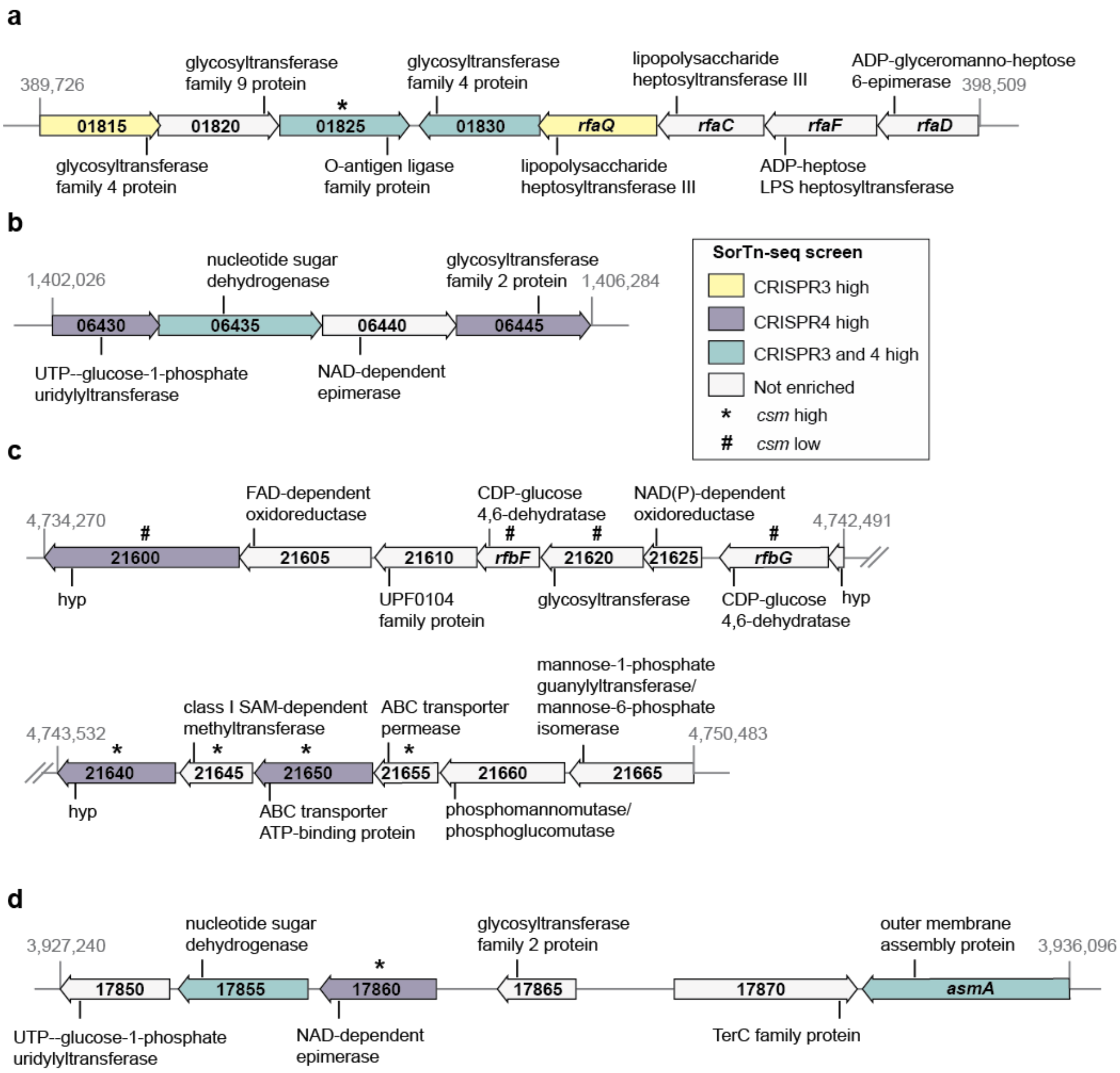

Figure S2. Operons involved in LPS/O-antigen synthesis and phospholipid were enriched during type III-A SorTn-seq. a. Operon predicted to be involved in synthesis of core LPS oligosaccharide components**. b.** Operon predicted to be involved polysaccharide biosynthesis. **c.** Operon predicted to be involved in LPS and O-antigen biosynthesis/transport. **d.** Operon predicted to be involved in phospholipid transport. Genes in **a-d** are colour coded by whether they were enriched in CRISPR3 high (yellow), CRISPR4 high (purple), both CRISPR3 and CRISPR4 (green), or not enriched (grey). Genes found in the *csm* (*cas10*) SorTn-seq are indicated by an asterisks (*) or hash (#), if enriched in the high or low, respectively.

a

LrhA *Eschericia coli* K12 (P36771)
HexA *Pectobacterium carotovorum* (O85272)
PecT *Dickeya dadantii* (P52662)
PigU *Serratia sp. ATCC 39006*

	LrhA	HexA	PecT	PigU
**LrhA**		66.33%	64.08%	64.08%
**HexA**	66.33%		82.33%	83.67%
**PecT**	64.08%	82.33%		87.70%
**PigU**	64.08%	83.67%	87.70%	

b

```
                     HTH LysR-type domain
LrhA MISANRPIINLDLDLLRTFVAVADLNTFAAAAAAVCRTQSAVSQQMQRLEQLVGKELFAR  60
HexA MTSANRPVLNLDLDLLRTFVAVADLNTFAAAATAVNRTQSAVSQQMQRLEQLIGKELFAR  60
PecT MTNTSRPVLNLDLDLLRTFVAVADLNTFAAAAVAVCRTQSAVSQQMQRLEQLIGKELFAR  60
PigU MTNANRPVLNLDLDLLRTFVAVADLNTFAAAAVAVCRTQSAVSQQMQRLEQLIGKELFAR  60
     * .:.**::***********************.** ****************:*******

LrhA HGRNKLLTEHGIQLLGYARKILRFNDEACSSLMFSNLQGVLTIGASDESADTILPFLLNR  120
HexA HGRNKLLTEHGIQFLGYARKILQFNDEACISLMYSDIQGTLTIGASDDTADTILPYILHR  120
PecT HGRNKLLTEHGIQFLGYARKILQFNDEACASLMYSDIQGTLTIGASDDTADTILPFILQR  120
PigU HGRNKLLTEHGIQFLNYARKILQFNDEACASLMYSDIQGVLTIGASDDTADTILPFILQR  120
     *************:*.******:****** ***:*::**.*******::******::*:*

LrhA VSSVYPKLALDVRVKRNAYMAEMLESQEVDLMVTTHRPSAFKALNLRTSPTHWYCAAEYI  180
HexA VTSVFPKLSVNVSVKRSAEMMEMLNQGKIDLVITTMNGVVFPHVLLRSSPTLWYCAADYQ  180
PecT VTNVFPKLSIAVSIKRSAEMTDMLQQGKIDLVITTSNNDDLPHVLLRTSPTLWYCSADYQ  180
PigU VTTVFPKLSVEVSIKRGTEMMDMLQEGKIDLIITTVNNTDLPHVLLRTSPTLWYCAADYQ  180
     *:.*:***:: * :**.: * :**:. ::**::** .   :  : **:*** ***:*:*

LrhA LQKGEPIPLVLLDDPSPFRDMVLATLNKADIPWRLAYVASTLPAVRAAVKAGLGVTARPV  240
HexA FRSQEPVPLVVLDEPSPFRTLATQQLTAAGIPWRIAYVASTLSAVRAAVKAGMGITVRSV  240
PecT YQPGETVSLVVLDEPSPFRALALDQLTAAGIPWKISYVASTLSAVRAAVKAGLGITVRSV  240
PigU FQPGETVSLVLLDEPSPFRSLALEQLTAAGIPWKISYVASTLSAVRAAVKAGLGITVRSV  240
     :  * : **:**:***** :.   *. *.***:::****** *********:*:*.* *

LrhA EMMSPDLRVLSGVDGLPPLPDTEYLLCYDPSSNNELAQVIYQAMESYHNPWQYSPMSAPE  300
HexA EMMSPELRVLGEEEGLPRLPETRYFLCQNPNQENELATAIFNVIESGKPSHIT-PVSMLA  299
PecT EMMSPELRVLGEEEGLPKLPDTRYYLCRNPDHDNELTNAIFSAIESGTRSHLL-PVSTGT  299
PigU EMMSPELRVLGEEEGLPRLPDTRYYLCKNPQHENELSTALFNAIESGARSHLL-PVGMLP  299
     *****:****.  :*** **:*.* ** :*. :***: .::..:**        *:.

LrhA GDDSLLIERDIE------- 312
HexA N------------------ 300
PecT E--SELREPPTDESLKDIT 316
PigU N--NDVREPPLE------- 309
```

Figure S3. PigU is similar to other characterised LysR-type transcriptional regulators. a. The *Serratia* PigU protein sequence was compared to similar proteins (as % amino acid identity) that have been experimentally characterised from *E. coli* K12 (LrhA), *Pectobacterium carotovorum* (formerly *Erwinia carotovorum* subsp. *carotovorum*) (HexA), *Dickeya dadantii* (formerly *Erwinia chrysanthemi*) (PecT). **b.** Alignment of PigU protein homologues (HexA, PecT, LrhA). These proteins contain a LysR-type Helix-Turn-Helix (HTH) domain as predicted by PROSITE (PS50931)

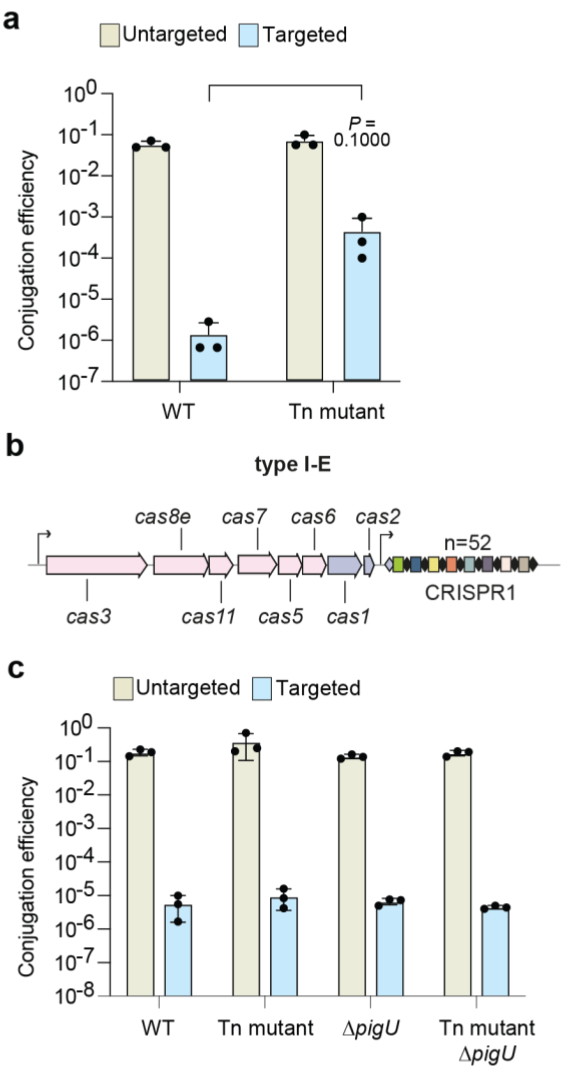

Figure S4. Increased PigU affects type III (CRISPR4) interference but has no effect on type I-E interference. a. CRISPR conjugation interference assay of an untargeted plasmid (pPF781) or a plasmid targeted (pPF3932) by CRISPR4 spacer 1 in the type III-A system in WT (LacA) and *pigU*pro transposon mutant (Tn mutant, HSPIG43). Statistical significance assessed using a Mann-Whitney test. **b.** Schematic of the type I-E CRISPR-Cas locus in *Serratia*. Interference genes are indicated in pink, adaptation genes in mauve and promoters as black arrows. The CRISPR1 array contains 52 spacers and is depicted with repeats (diamonds) and spacers (coloured rectangles). **c.** Conjugation efficiency of an untargeted plasmid (pPF719) or a plasmid targeted (pPF724) by the type I-E system in WT (LacA), a *pigU*pro transposon mutant (Tn mutant, HSPIG43), a Δ*pigU* mutant (Δ*pigU*, PCF720) and a *pigU*pro, Δ*pigU* double mutant (Tn mutant Δ*pigU*, PCF707). Statistical significance of conjugation efficiency (targeted strains) assessed using a one-way ANOVA (P=0.4398). Bars are the means and error bars +/- SD. Individual biological replicates are shown (n=3).

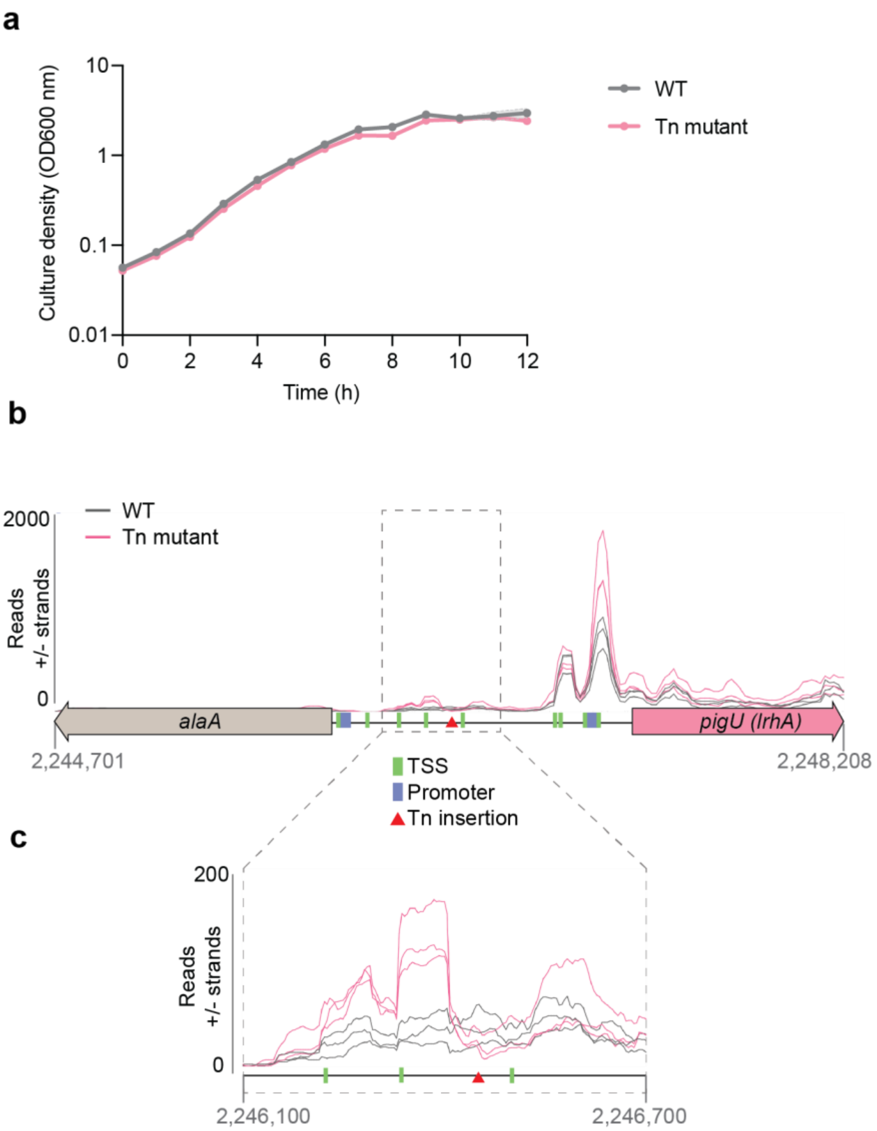

Figure S5. Growth during RNA extraction and RNA-seq reads mapped to the *alaA-pigU* genes and intergenic region. a. Culture density (optical density OD at 600 nm) was measured for the WT (LacA) and *alaA-pigU* transposon mutant (HSPIG43) over the course of 12 h. RNA was extracted at early stationary phase (12 h), where the WT mean OD600=2.936 (SD 0.3523), and Tn mutant mean OD600=2.420 (SD 0.1493). Solid lines represent the mean and shaded areas represent the standard deviation (*n*=3). **b.** Transcription of *pigU* is increased in the transposon mutant compared to the WT. **c.** Zoom in of dashed region in **a** focusing on reads around the transposon insertion site. For **b** and **c**, promoter sequence = blue rectangles, transcription start sites = green rectangles, transposon insertion site = red triangle; reads are indicated by pink (transposon mutant) and grey (WT) lines. Individual biological replicates are shown (*n*=3).

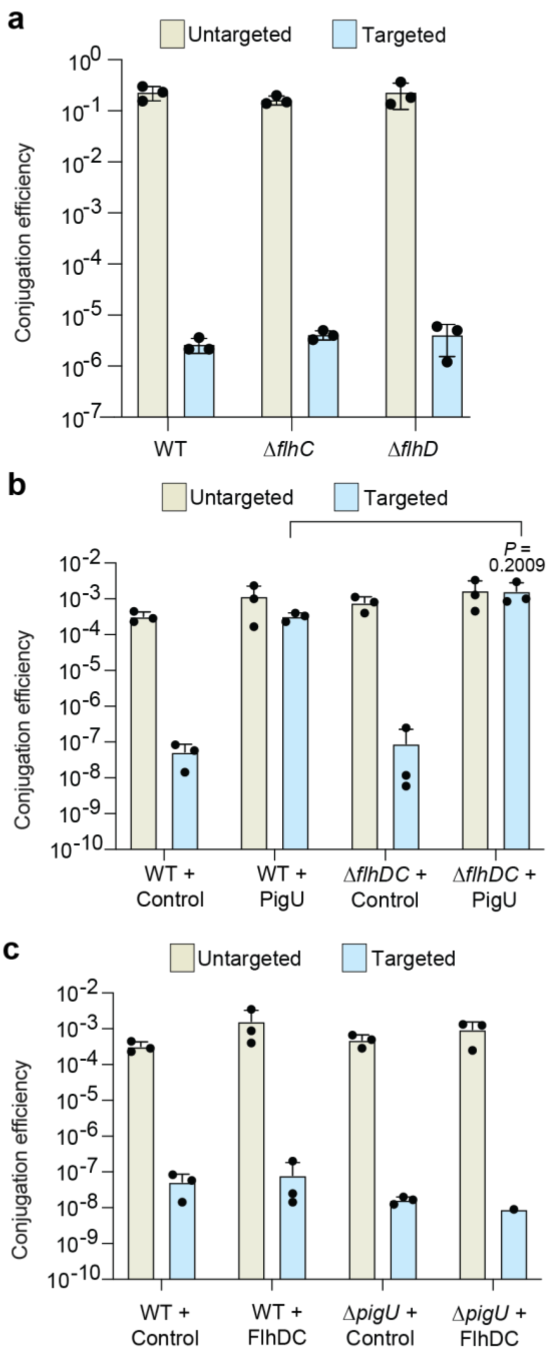

Figure S6. Changes in flagella do not contribute to PigU-mediated CRISPR-Cas repression. a. CRISPR conjugation interference assay of an untargeted plasmid (pPF781) or a plasmid targeted (pPF1043) by the type III-A system in WT (PCF396), a Δ*flhC* mutant (PCF626), and a Δ*flhD* mutant (PCF627). Statistical significance of conjugation efficiency (targeted strains) assessed using a one-way ANOVA (*P*= 0.4920). **b.** CRISPR conjugation interference assay of an untargeted plasmid (pPF1621) or a plasmid targeted (pPF2841) the type III-A system in WT (LacA) or a Δ*flhDC* mutant (PCF879) containing a vector control (pQE-80LoriT; Control) or a plasmid expressing PigU (pPF1983; PigU). Statistical significance assessed using a two-tailed Student's t-test with Welch's correction. **c.** CRISPR conjugation interference assay as in (b) in WT (LacA) or a Δ*pigU* mutant (PCF720) containing a vector control (pQE-80LoriT; Control) or a plasmid expressing FlhDC (pPF516; FlhDC). Statistical significance assessed using a two-tailed Student's t-test with Welch's correction. In (a), (b) and (c), bars are the means and error bars SD; individual biological replicates are shown (*n*=3). In (c), for Δ*pigU* + FlhDC (targeted), conjugation efficiency could not be calculated for two replicates as cells harbouring the targeted plasmid fell below the limit of detection of the assay (<100 CFU/ml).

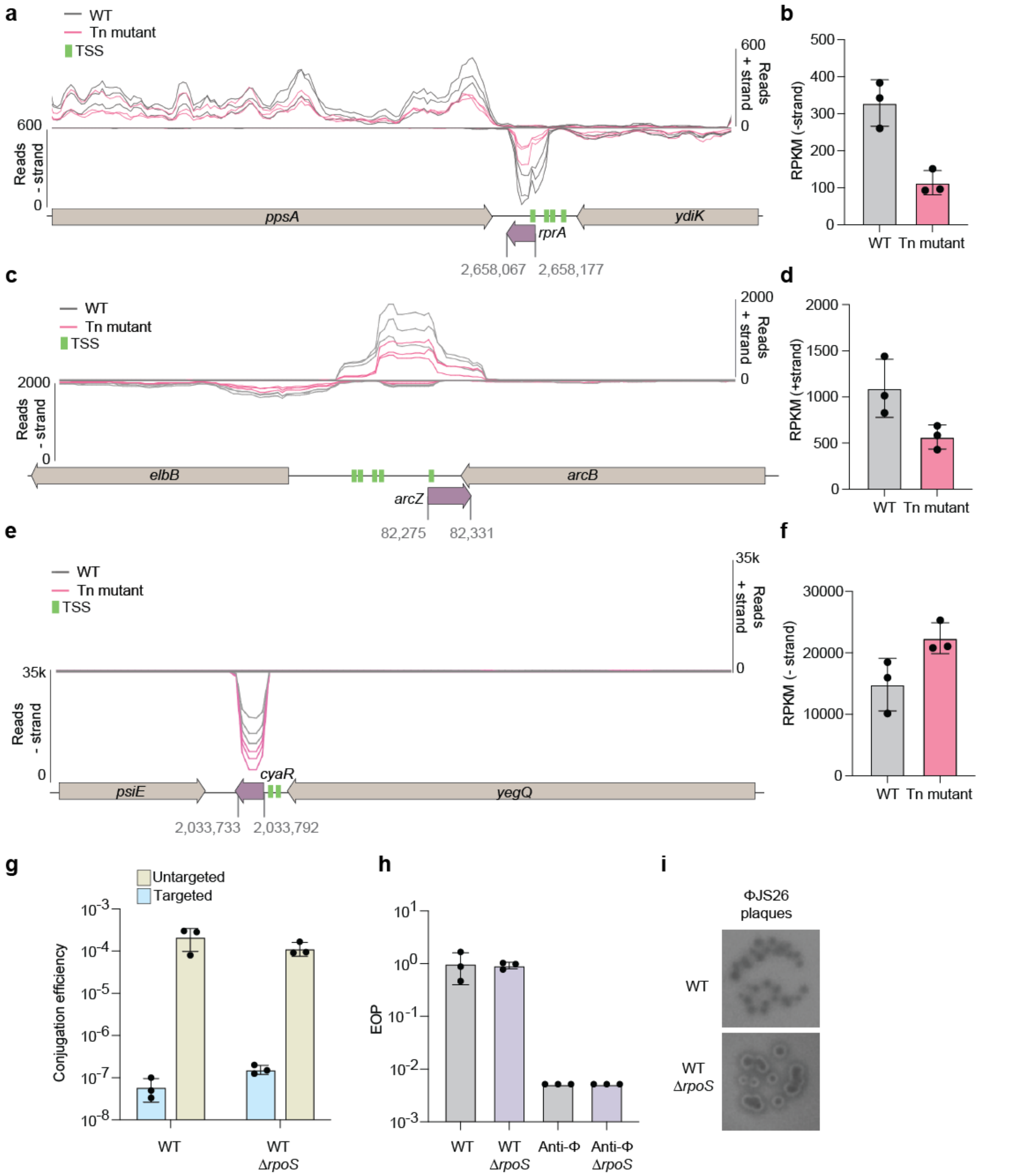

Figure S7. sRNAs controlling the transcription and translation of *rpoS* are downregulated during *pigU* overexpression. a. Read mapping in the *rprA* gene region. **b.** Reads Per Kilobase of transcript per Million mapped reads (RKPM) of the *rprA* transcript. **c.** Read mapping in the predicted *arcZ* gene region. **d.** RKPM of the *arcZ* transcript. **e.** Read mapping in the predicted *cyaR* gene region **f.** RKPM of the predicted *cyaR* transcript. **g.** Type III-A conjugation efficiency of the WT (LacA) versus the *rpoS* mutant (WT Δ*rpoS*, NMW25). **h.** Quantification of the efficiency of plating (EOP), relative to the WT (LacA), for the *rpoS* mutant (WT Δ*rpoS*, NMW25), type III-A ΦJS26 targeting strain (Anti-Φ, PCF925), and type III-A JS26 targeting strain with *rpoS* deletion (Anti-Φ Δ*rpoS*, PCF944). Bars are the means and error bars +/- SD. Individual biological replicates are shown (*n*=3). **i.** JS26 plaque phenotypes for the WT (LacA) and *rpoS* mutant (NMW25). For **a**, **c**, and **e**, reads are indicated by pink (transposon mutant) and grey (WT) lines. Individual biological replicates are shown (*n*=3). Transcriptional start sites (TSS; green). For **b, d,** and **f**, RPKMs were calculated from the furthest upstream transcription start site to the 3' end of the predicted gene location.

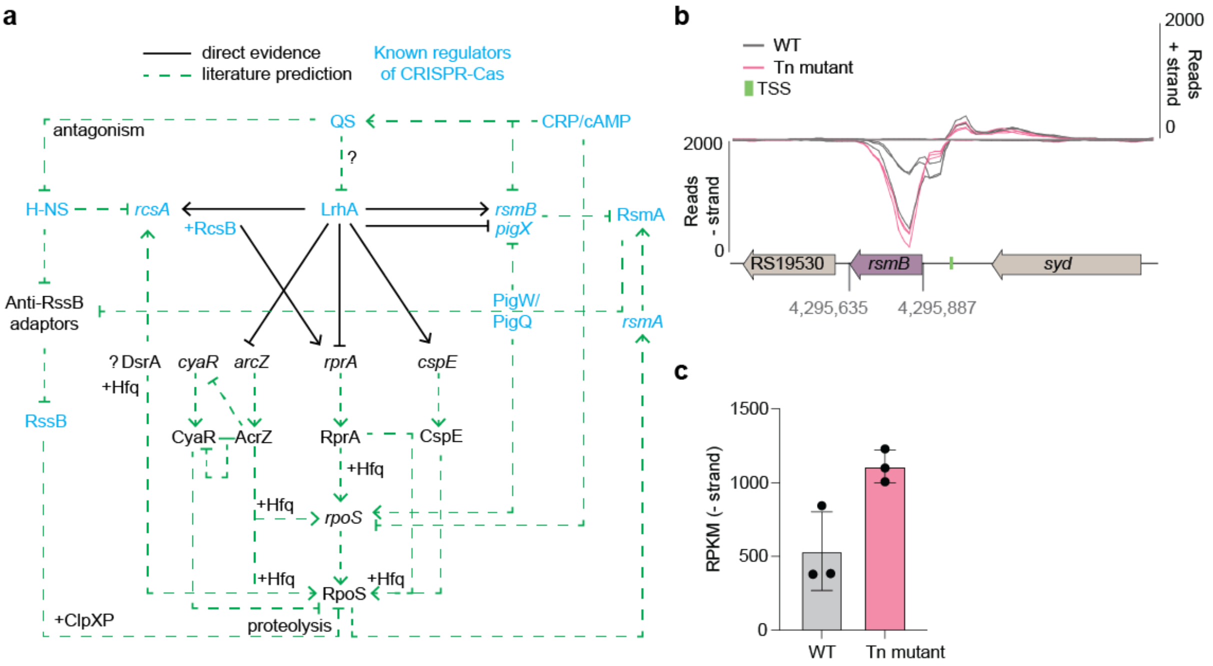

Figure S8. Genes under control of PigU overlap with the regulons of several known CRISPR-Cas regulators. a. Connections between the PigU regulon and other known CRISPR-Cas regulators. Direct evidence from this study (solid black lines), predictions from the literature (green dashed lines) (2-18)**,** known CRISPR-Cas regulators (blue) (19-27). **b.** Read mapping in the *rsmB* gene region. This gene is not included in the *Serratia* sp. ATCC 39006 annotation and was identified through previous Rsm pathway analysis (20). **c.** Reads Per Kilobase of transcript per Million mapped reads (RKPM) of the *rsmB* gene, as calculated from the furthest upstream transcription start site to the 3' end of the predicted gene location For **b** and **c**, reads are indicated by pink (transposon mutant) and grey (WT) lines.

Table S1. Bacterial strains used in this study.

Name	Genotype/Phenotype	Reference
Escherichia coli DH5α	Cloning strain: F^-, ϕ80Δd*lacZ*M15, Δ(*lacZYA–argF*)U169, *endA1, recA1, hsd*R17 (r_K^- m_K^+), *deoR*, *thi*-1, *sup*E44, λ^-, *gyrA*96, *relA*1	Gibco/BRL
E. coli ST18	Conjugative donor: *pro,hsdR, recA*::RP4-2-Tc::Mu, Tmp^R, Sp^R, Sm^R, λ*pir* Δ*hemA*	(28)
Serratia sp. ATCC 39006 LacA	Lac^- mutant generated by EMS mutagenesis	(29,30)
Serratia LacA-derivatives		
HSPIG43	*pigU*pro::mini-Tn5*lacZ1*, Km^R	(31)
NMW25	*rpoS::Tn-uidA,* Cm^R	(32)
PCF396	Δ*pigA-O*, unmarked	(33)
PCF632	*pigU*pro::mini-Tn5*lacZ1*, Δ*pigA-O*, Km^R	This study
PCF707	*pigU::uidA*, *pigU*pro::mini-Tn5*lacZ1*, Cm^R Km^R	This study
PCF708	*pigU::uidA*, Δ*pigA-O*, Cm^R	This study
PCF709	*pigU::uidA*, *pigU*pro::mini-Tn5*lacZ1*, Δ*pigA-O*, Cm^R Km^R	This study
PCF720	*pigU::uidA*, Cm^R	(34)
PCF879	Δ*flhDC*	(35)
PCF525	Spacer in CRISPR2 (type I-F) targeting phage JS26 (JT354_gp61 encoding Tape Measure Protein)	(36)
PCF925	Spacer in CRISPR3 (type III-A) targeting phage JS26 (JT354_gp01 encoding hypothetical protein)	This study
PCF944	Spacer in CRISPR3 (type III-A) targeting phage JS26 (JT354_gp01 encoding hypothetical protein), *rpoS::Tn-uidA,* Cm^R	This study

Table S2. Plasmids used in this study. Multiple cloning site, MCS; origin of replication, ori; origin of transfer, oriT. Chloramphenicol, Cm; Ampicillin, Ap, Gentamicin, Gm, Tetracycline, Tc.

Name	Description	Construction	Reference
CRISPR and cas promoter-eYFP reporters			
pPF1307	eYFP only FACS gating control<br>Type III-A *cas10* promoter eYFP, *lacI*/T5*lac*, RP4 oriT, pBR322 ori, Cm^R		(33)
pPF1438	mCherry only FACS gating control<br>Empty vector, mCherry, *lacI*/T5*lac*, RP4 oriT, pBR322 ori, Cm^R		(33)
pPF1439	eYFP, mCherry, *lacI*/T5*lac*, RP4 oriT, pBR322 ori, Cm^R		(33)
pPF1567	CRISPR promoter expression vector<br>Empty vector, eYFP mCherry *lacI*/T5*lac*, RP4 oriT, pBR322 ori, Cm^R	Annealed 3 frame stop + RBS + enhancer region (primers PF3095+PF3096) and ligated to pPF1439 (BamHI/NsiI) using T4 ligase	This study
pPF1923	Type III-A CRISPR3 promoter eYFP mCherry *lacI*/T5*lac*, RP4 oriT, pBR322 ori, Cm^R	PCR amplified CRISPR3 promoter from *Serratia* with primers PF3736+PF3737 and ligated into pPF1567 (SpeI/NsiI) using T4 ligase	This study
pPF1924	Type III-A CRISPR4 promoter eYFP mCherry *lacI*/T5*lac*, RP4 oriT, pBR322 ori, Cm^R	PCR amplified CRISPR3 promoter from *Serratia* with primers PF3738+PF3739 and ligated into pPF1567 (SpeI/NsiI) using T4 ligase	This study
Transposon mutagenesis			
pKRCPN2	Conjugative plasmid containing Tn-DS1028*uidA*Km (mini-Tn5-based), R6K ori, Km^R, Tc^R		(37,38)
Expression plasmids			
pQE-80LoriT	IPTG-inducible expression vector, *lacI/T5*, pBR322 ori, RP4 oriT, Ap^R		(39)
pPF1983	PigU expression vector (pQE-80LoriT-derivative); *lacI/T5*, pBR322 ori, RP4 oriT, Ap^R	PCR amplified the *pigU* gene from *Serratia* (CWC46_RS10440) with primers PF794 + PF795 and ligated into pQE-80LoriT (EcoRI and HindIII) using T4 ligase	This study
pPF516	FlhDC expression vector (pQE-80LoriT-derivative); *lacI/T5*, pBR322 ori, RP4 oriT, Ap^R		(35)
Conjugation interference assays			
pPF781	Untargeted control for the type III-A system, p15a ori, RP4 oriT, araC, Cm^R		(21)
pPF1043	Targeted type III-A CRISPR3 spacer 1, p15a ori, RP4 oriT, araC, Cm^R		(21)
pPF1621/ pSEVA6210	Untargeted control for the type III-A system, RK2 ori, RP4 oriT, araC, Gm^R		(40)
pPF2841	Targeted type III-A CRISPR3 spacer 1, RK2 ori, oriT, araC, Gm^R	Annealed primers PF2086 and PF2087 and ligated into KpnI/SphI digested pPF1621	This study

Name	Description	Construction	Reference
pPF3932	Targeted type III-A CRISPR4 spacer 1, RK2 ori, oriT, araC, Gm^R	Annealed primers PF8122 and PF8123 and ligated into KpnI/SphI digested pPF781	This study
pPF719	Untargeted control for type I-E and I-F systems, pQE-80LoriT-GFP, Tc^R		(21)
pPF724	Targeted type I-E CRISPR1 spacer 1 (CTT PAM) pQE-80LoriT-GFP-derivative, Tc^R		(21)
pPF722	Targeted type I-F CRISPR2 spacer 1 (GG PAM) pQE-80Lori-T-GFP-derivative, Tc^R		(21)
CRISPR adaptation assays			
pPF781	p15a ori, RP4 oriT, araC, Cm^R		(21)
pPF3804	Entry vector mCherry, p15a ori, RP4 oriT, araC, Cm^R	PCR of pPF1438 template with primers PF7821/PF7822. Product Gibson assembled into pPF781(SacI/PstI)	This study
pPF3805	Priming control vector mCherry, BsaI, p15a ori, RP4 oriT, araC, Cm^R	Digest of pPF3804 (XmaI) Gibson assembled with annealed primers PF7823/PF7824	This study
pPF3807	Priming type I-F CRISPR2 spacer 2 (AGA PAM) mCherry, p15a ori, RP4 oriT, araC, CmR	Digest of pPF3805 (BsaI) ligated with annealed primers PF7827/PF7828 using T4 ligase	This study
Allelic exchange mutagenesis			
pPF976	*lacI*/T5*lac*, RP4 oriT, pBR322 ori, Km^R	Type III-A repeat-BsaI-repeat construct for artificial crRNA	(41)
pPF1117	R6K ori, RP4 oriT, Cm^R, SacB	Suicide vector for chromosomal allelic exchange	(42)
pPF3304	CRISPR3 upstream/downstream flanks, R6K ori, RP4 oriT, Cm^R, SacB	CRISPR3 flanks amplified from *Serratia* (PF6491 + PF6509 and PF6510 + PF6552) and mini-array repeat-BsaI-repeat amplified from pPF976 (PF6508 + PF6511) Gibson assembled into pPF1117 (BamHI/NsiI)	This study
pPF3581	Mini-CRISPR array (repeat-anti J626 JT354_gp01 spacer-repeat), CRISPR3 upstream/downstream flanks, R6K ori, RP4 oriT, Cm^R, SacB	pPF3304 digested with BsaI, PF7082 + PF7083 annealed, ligated with T4 ligase	This study

Table S3. Oligonucleotides used in this study.

Name	Description	Sequence	Notes
Reporter plasmids			
PF3095	Fw 3x STOP enhancer RBS	TCTAGGTACCCCTAAATGATTAACTTTATAA GGAGGAAAAACTAATGG	Kpn1 site, 3x stop, T7 enhancer, RBS, ATG
PF3096	Rv 3x STOP enhancer RBS	GATCCCATTAGTTTTTCCTCCTTATAAAGTT AATCATTTAGGGGTACCTAGATGCA	NsiI overhang Kpn1 site, 3x stop, T7 enhancer, RBS, ATG, BamHI overhang
PF3736	Fw CRISPR3 promoter	TTTACTAGTCTCTGAACCCGCCACC	SpeI
PF3737	Rv CRISPR3 promoter	TTTATGCATCTTCAGTCAGGGAGCGTC	NsiI
PF3738	Fw CRISPR4 promoter	TTTACTAGTCCGCGATATCAGCCC	SpeI
PF3739	Rv CRISPR4 promoter	TTTATGCATCAGTCAGGGAACGTCCTTAC	NsiI
PigU expression plasmid			
PF794	Fw *pigU*	ATAGAATTCAGGAGGAATATAATGACAAATG CAAATCGCC	KpnI/SphI sites
PF795	Rv *pigU*	GATAAGCTTTTACTCTAAAGGTGGTTCCCG	
Conjugation interference plasmids			
PF2086	Oligo to anneal protospacer targeted by *Serratia* III-A CRISPR3 spacer 1	CTTGGAAAAAAACCGACACGTAGTGTGAAAG AAATTAGGATGAGCATG	KpnI/SphI overhangs
PF2087	Oligo to anneal protospacer targeted by *Serratia* III-A CRISPR3 spacer 1	CTCATCCTAATTTCTTTCACACTACGTGTCG GTTTTTTTCCAAGGTAC	KpnI/SphI overhangs
PF8122	Oligo to anneal protospacer targeted by *Serratia* III-A CRISPR4 spacer 1	CTTGGTATAGATTGTGGGGATACATATCCAG ATAATAAATGAGCATG	KpnI/SphI overhangs
PF8123	Oligo to anneal protospacer targeted by *Serratia* III-A CRISPR4 spacer 1	CTCATTTATTATCTGGATATGTATCCCCACA ATCTATACCAAGGTAC	KpnI/SphI overhangs
Adaptation priming plasmids			
PF7821	Fw mCherry	GGGCTAGCGAATTCGAGCTCAGGAGGAATTA ACATGGTGAGCAAGGGC CAAAAGGTCATCCACTGCAGTTACTTGTACA GCTC	Gibson overlaps
PF7822	Rv mCherry	CAAAAGGTCATCCACTGCAGTTACTTGTACA GCTC	Gibson overlaps
PF7823	Oligo to anneal BsaI sites	CAGCGGAAAAGCGCTCCCGGGGAGACCTGCT GACGACGAGGACGGTCTCC	Gibson overlaps
PF7824	Oligo to anneal BsaI sites	AGTATACACTCCGCTAGCGCCCGGGGAGACC GTCCTCGTCGTCAGCAGGTCTCC	Gibson overlaps
PF7827	Oligo to anneal priming protospacer (*Serratia* I-F CRISPR2 spacer 2 AGA PAM)	CCGGTCTTGGAAGCCGCCAGTGTCGTCAGCA TCAGATGT	BsaI overhangs
PF7828	Oligo to anneal priming protospacer (*Serratia* I-F CRISPR2 spacer 2 AGA PAM)	CCGGACATCTGATGCTGACGACACTGGCGGC TTCCAAGA	BsaI overhangs

Name	Description	Sequence	Notes
Allelic exchange CRISPR3			
PF6491	Fw CRISPR3 upstream flank	CTGCAGGTCGACCGCTAGCTGGATCCCGGCTGCGAAAAATGC	Gibson overlaps
PF6509	Rv CRISPR3 upstream flank	CAGGGAGCGTCCGTAAGGACAATGAAAATTTATAACCCAT	Gibson overlaps
PF6510	Fw CRISPR3 downstream flank	CTGACTGAAGGGATTAAGACTCCACCGGACGGTTTTCCGC	Gibson overlaps
PF6552	Rv CRISPR3 downstream flank	TTGGCCCAGGGCTTCCCGGTATGCATATCGACCCCCAGCGGGGC	Gibson overlaps
PF6508	Fw repeat-BsaI-repeat	ATGGGTTATAAATTTTCATTGTCCTTACGGACGCTCCCTG	Gibson overlaps
PF6511	Rv repeat-BsaI-repeat	GCGGAAAACCGTCCGGTGGAGTCTTAATCCCTTCAGTCAG	Gibson overlaps
PF7082	Oligo to anneal anti-JS26 JT354_gp01 III-A spacer	ACACAACGACATACAGCAGGCCGCGCGGATTCACTTTTG	BsaI overhangs
PF7083	Oligo to anneal anti-JS26 JT354_gp01 III-A spacer	AGGACAAAAGTGAATCCGCGCGGCCTGCTGTATGTCGTT	BsaI overhangs
Screening / sequencing			
PF209	Fw pQE-80LoriT MCS	TCGTCTTCACCTCGAGAAATC	
PF210	Rv pQE-80LoriT MCS	GTCATTACTGGATCTATCAACAGG	
PF796	Fw RNA-seq DNA contamination check	ATAGAATTCAGGAGGAATATAATGGGTACTTCTGAGTTACTTAAGC	
PF797	Rv RNA-seq DNA contamination check	GATCCCGGGTCAGACTGCGTGTTTTACTTG	
PF2165	Fw allelic exchange vectors	CTCATTTCACTAAATAATAGTGAACG	
PF5194	Rv allelic exchange vector/adaptation vectors	CCTCTTACGTGCCGATCAAC	
PF3252	Fw CRISPR3 spacer knock in	TTTTGAATTCGATTTATGGCAACAGCACCAG	
PF3816	Rv CRISPR3 spacer knock	GGGCTTCCCGGTATGCATGCGTCATCCACAGGCTGTCGAG	
PF138	Fw adaptation vectors	CACACTTTGCTATGCCATAG	
PF7829	Fw adaptation protospacer screening	GTCTACACGAACCCTTTG	
PF7830	Rv adaptation protospacer screening	CACATATTCTGCTGACGC	
PF1888	Fw CRISPR2 (I-F) leader	CATCTGATGCTGACGACACTG	
PF1990	Rv CRISPR2 (I-F) spacer 2	CACGAAAATGATAATTGATGCTGAT	
Transposon mutagenesis sequencing library construction			
PF3139	SorTn-seq 1st round enrichment primer, binds Tn*5*,-s- indicates phosphorothioate bond	/5BiotinTEG/TCATCTGCAGCCGGGAATTCTCATGTTTGACA-s-G	pair with PF3140; contains 5' biotin with a triethyleneglycol (TEG) spacer

Name	Description	Sequence	Notes
PF3140	SorTn-seq 1st round enrichment primer, binds NEBNext® adapter for Illumina®; -s- indicates phosphorothioate bond	GTGACTGGAGTTCAGACGTGTGCTCTTCCGATC-s-T	pair with PF3139
PF3270	SorTn-seq 2nd round enrichment primer, binds Tn*5* and adds Illumina P5 sequence;, -s- indicates phosphorothioate bond	AATGATACGGCGACCACCGAGATCTACACCGCGCGATAAATCTAGAGTCGACCT-s-G	pair with Illumina indexing primer
PF3124	Illumina P5 site qPCR primer	AATGATACGGCGACCACCGAG	pair with PF3125
PF3125	Illumina P7 site qPCR primer	CAAGCAGAAGACGGCATACGA	pair with PF3124
PF2926	SorTn-seq custom sequencing primer, binds transposon downstream of PF3270	CAGGCATGCAAGCTTCAGGGTTGAGATGTG	
PF3441	Illumina Read 1 primer, for sequencing PhiX library	ACACTCTTTCCCTACACGACGCTCTTCCGATCT	
NEBNext Index 1 Primer for Illumina	Index sequence underlined, -s- indicates phosphorothioate bond	CAAGCAGAAGACGGCATACGAGAT**CGTGAT**GTGACTGGAGTTCAGACGTGTGCTCTTCCGATC-s-T	NEBNext® Multiplex Oligos for Illumina® (Index Primers Set 1)
NEBNext Index 2 Primer for Illumina	Index sequence underlined, -s- indicates phosphorothioate bond	CAAGCAGAAGACGGCATACGAGAT**ACATCG**GTGACTGGAGTTCAGACGTGTGCTCTTCCGATC-s-T	NEBNext® Multiplex Oligos for Illumina® (Index Primers Set 1)
NEBNext Index 3 Primer for Illumina	Index sequence underlined, -s- indicates phosphorothioate bond	CAAGCAGAAGACGGCATACGAGAT**GCCTAA**GTGACTGGAGTTCAGACGTGTGCTCTTCCGATC-s-T	NEBNext® Multiplex Oligos for Illumina® (Index Primers Set 1)
NEBNext Index 4 Primer for Illumina	Index sequence underlined, -s- indicates phosphorothioate bond	CAAGCAGAAGACGGCATACGAGAT**TGGTCA**GTGACTGGAGTTCAGACGTGTGCTCTTCCGATC-s-T	NEBNext® Multiplex Oligos for Illumina® (Index Primers Set 1)
NEBNext Index 5 Primer for Illumina	Index sequence underlined, -s- indicates phosphorothioate bond	CAAGCAGAAGACGGCATACGAGAT**CACTGT**GTGACTGGAGTTCAGACGTGTGCTCTTCCGATC-s-T	NEBNext® Multiplex Oligos for Illumina® (Index Primers Set 1)
NEBNext Index 6 Primer for Illumina	Index sequence underlined, -s- indicates phosphorothioate bond	CAAGCAGAAGACGGCATACGAGAT**ATTGGC**GTGACTGGAGTTCAGACGTGTGCTCTTCCGATC-s-T	NEBNext® Multiplex Oligos for Illumina® (Index Primers Set 1)

Name	Description	Sequence	Notes
NEBNext Index 7 Primer for Illumina	Index sequence underlined, -s- indicates phosphorothioate bond	CAAGCAGAAGACGGCATACGAGAT**GATCTG**G TGACTGGAGTTCAGACGTGTGCTCTTCCGAT C-s-T	NEBNext® Multiplex Oligos for Illumina® (Index Primers Set 1)
NEBNext Index 8 Primer for Illumina	Index sequence underlined, -s- indicates phosphorothioate bond	CAAGCAGAAGACGGCATACGAGAT**TCAAGT**G TGACTGGAGTTCAGACGTGTGCTCTTCCGAT C-s-T	NEBNext® Multiplex Oligos for Illumina® (Index Primers Set 1)
NEBNext Index 9 Primer for Illumina	Index sequence underlined, -s- indicates phosphorothioate bond	CAAGCAGAAGACGGCATACGAGAT**CTGATC**G TGACTGGAGTTCAGACGTGTGCTCTTCCGAT C-s-T	NEBNext® Multiplex Oligos for Illumina® (Index Primers Set 1)
NEBNext Index 10 Primer for Illumina	Index sequence underlined, -s- indicates phosphorothioate bond	CAAGCAGAAGACGGCATACGAGAT**AAGCTA**G TGACTGGAGTTCAGACGTGTGCTCTTCCGAT C-s-T	NEBNext® Multiplex Oligos for Illumina® (Index Primers Set 1)
NEBNext Index 11 Primer for Illumina	Index sequence underlined, -s- indicates phosphorothioate bond	CAAGCAGAAGACGGCATACGAGAT**GTAGCC**G TGACTGGAGTTCAGACGTGTGCTCTTCCGAT C-s-T	NEBNext® Multiplex Oligos for Illumina® (Index Primers Set 1)
NEBNext Index 12 Primer for Illumina	Index sequence underlined, -s- indicates phosphorothioate bond	CAAGCAGAAGACGGCATACGAGAT**TACAAG**G TGACTGGAGTTCAGACGTGTGCTCTTCCGAT C-s-T	NEBNext® Multiplex Oligos for Illumina® (Index Primers Set 1)
NEBNext Adaptor for Illumina	-/5Phos/ indicates 5' phosphate, -s- indicates phosphorothioate bond	-/5Phos/GAT CGG AAG AGC ACA CGT CTG AAC TCC AGT CdUA CAC TCT TTC CCT ACA CGA CGC TCT TCC GAT C-s-T	NEBNext® Multiplex Oligos for Illumina® (Index Primers Set 1)

Supplementary Excel Files

Table S4. SorTn-seq mapping and insertion summary. Total reads are the number following de-multiplexing. Reads with Tn*5* tag and % with Tn*5* tag are those containing the 12 nt transposon 'tag' generated during sequencing (2 mismatches allowed). Reads mapped and % mapped are those mapping to the *Serratia* LacA genome (RefSeq: NZ_CP025085.1) using the TraDIS pipeline(43). Unique insertion sites (UIS) = the total number of unique insertions. Length/UIS is the average number of unique insertions per nt for the *Serratia* genome (4.97 MB).

Table S5. Features enriched in CRISPR3 SorTn-seq. Features (gene or intergenic) are those significantly enriched in either the low or high sort bin (as compared to the depleted control), with a log2 fold change > 0.5 and P < 0.05 (Benjamini and Hochberg method correction) as calculated via *edgeR*(44). Gene annotations and nucleotide positions (start and end) obtained from the *Serratia* sp. ATCC 39006 General Feature Format annotation (.gff) (RefSeq: NZ_CP025085.1).

Table S6. Features enriched in CRISPR4 SorTn-seq. Features (gene or intergenic) are those significantly enriched in either the low or high sort bin (as compared to the depleted control), with a log2 fold change > 0.5 and P < 0.05 (Benjamini and Hochberg method correction) as calculated via *edgeR*(44). Gene annotations and nucleotide positions (start and end) obtained from the *Serratia* sp. ATCC 39006 General Feature Format annotation (.gff) (RefSeq: NZ_CP025085.1).

Table S7. SorTn-seq enrichment comparisons. Features (gene or intergenic) shared between the different type III-A SorTn-seq (CRISPR3, CRISPR4, *csm*) by sort bin (high or low).

Table S8. Differentially expressed genes in the *alaA-pigU* transposon mutant. Genes shown are significantly different from the wildtype control, with a | log2 fold change > 0 | and adjusted p-value < 0.05 (Benjamini and Hochberg method correction), as calculated by DESeq2 (45).

Table S9. Toxin-antitoxin (TA) systems differentially expressed in the transposon mutant. TA-systems based on characterization performed in (30). FC (log2), log2 fold change from DeSeq2 analysis; p-value (adj.), adjusted p-value from DESeq2 analysis; NS, not significant.

TA system	Toxin locus tag	FC (log2)	p-value (adj.)	Antitoxin locus tag	FC (log2)	p-value (adj.)
Type I						
symE-7	CWC46_ RS17015	1.08	5.56E-03	-	-	-
Type II						
relBE/parD E-1	CWC46_ RS00060	1.21	1.70E-05	CWC46_ RS00065	0.89	3.99E-06
relBE/parD E-2	CWC46_ RS00090	0.61	1.36E-02	CWC46_ RS00095	0.73	2.44E-03
hicAB-1	CWC46_ RS06510	0.95	1.03E-06	CWC46_ RS06505	NS	NS
parDE-1	CWC46_ RS13005	1.05	7.38E-04	CWC46_ RS13000	1.44	1.02E-04
higAB-2	CWC46_ RS15595	0.71	4.25E-02	CWC46_ RS15590	NS	NS
hipAB-5	CWC46_ RS20605	-0.54	1.24E-02	CWC46_ RS20610	NS	NS
hicAB-2	CWC46_ RS14310	1.36	8.46E-08	CWC46_ RS14305	NS	NS
Type IV						
abiE-3	CWC46_ RS06670	NS	NS	CWC46_ RS06665	0.79	4.18E-02
Type V						
ghoT-1	CWC46_ RS11680	0.55	4.08E-02	-	-	-

Table S10. Genes significantly enriched in both type III-A SorTn-seq (*csm/cas10*, CRISPR3 or CRISPR4) and RNA-seq of the *alaA-pigU* intergenic transposon mutant.

Name	Locus tag	Feature	Start	End	Product
glnL	CWC46_RS02180	gene	473314	474363	nitrogen regulation protein NR(II)
CWC46_RS06525	CWC46_RS06525	pseudo gene	1417230	1417541	RNA-dependent DNA polymerase
CWC46_RS09790	CWC46_RS09790	gene	2109371	2109979	LuxR family transcriptional regulator (RcsA)
CWC46_RS09795	CWC46_RS09795	gene	2110045	2110740	EAL domain-containing protein
flhD	CWC46_RS11345	gene	2490576	2490926	flagellar transcriptional regulator FlhD
flhC	CWC46_RS11350	gene	2490932	2491510	flagellar transcriptional regulator FlhC
flhB	CWC46_RS11400	gene	2501781	2502932	flagellar type III secretion system protein FlhB
flgD	CWC46_RS11440	gene	2508040	2508714	flagellar hook assembly protein FlgD
CWC46_RS20795	CWC46_RS20795	gene	4562782	4563396	helix-turn-helix transcriptional regulator (PigP)
CWC46_RS21600	CWC46_RS21600	gene	4734270	4736231	hypothetical protein
rfbF	CWC46_RS21615	gene	4738596	4739372	glucose-1-phosphate cytidylyltransferase
CWC46_RS21620	CWC46_RS21620	gene	4739385	4740368	glycosyltransferase family 2 protein
CWC46_RS21640	CWC46_RS21640	gene	4743532	4744734	hypothetical protein
CWC46_RS21645	CWC46_RS21645	gene	4744780	4745592	class I SAM-dependent methyltransferase
CWC46_RS21650	CWC46_RS21650	gene	4745600	4746895	ABC transporter ATP-binding protein
CWC46_RS22535	CWC46_RS22535	gene	4925797	4926900	MFS transporter
CWC46_RS22970	CWC46_RS22970	gene	4740365	4741186	NAD-dependent epimerase/dehydratase family protein

References

1. Miller, W.G. and Lindow, S.E. (1997) An improved GFP cloning cassette designed for prokaryotic transcriptional fusions. *Gene*, **191**, 149-153.
2. Battesti, A., Majdalani, N. and Gottesman, S. (2011) The RpoS-mediated general stress response in Escherichia coli. *Annu Rev Microbiol*, **65**, 189-213.
3. Yuan, X., Zeng, Q., Khokhani, D., Tian, F., Severin, G.B., Waters, C.M., Xu, J., Zhou, X., Sundin, G.W., Ibekwe, A.M. *et al.* (2019) A feed-forward signalling circuit controls bacterial virulence through linking cyclic di-GMP and two mechanistically distinct sRNAs, ArcZ and RsmB. *Environ Microbiol*, **21**, 2755-2771.
4. Neubacher, N., Tobias, N.J., Huber, M., Cai, X., Glatter, T., Pidot, S.J., Stinear, T.P., Lutticke, A.L., Papenfort, K. and Bode, H.B. (2020) Symbiosis, virulence and natural-product biosynthesis in entomopathogenic bacteria are regulated by a small RNA. *Nat Microbiol*, **5**, 1481-1489.
5. Kim, W. and Lee, Y. (2020) Mechanism for coordinate regulation of rpoS by sRNA-sRNA interaction in Escherichia coli. *RNA Biol*, **17**, 176-187.
6. Peterson, C.N., Carabetta, V.J., Chowdhury, T. and Silhavy, T.J. (2006) LrhA regulates rpoS translation in response to the Rcs phosphorelay system in Escherichia coli. *J Bacteriol*, **188**, 3175-3181.
7. Majdalani, N., Chen, S., Murrow, J., St John, K. and Gottesman, S. (2001) Regulation of RpoS by a novel small RNA: the characterization of RprA. *Mol Microbiol*, **39**, 1382-1394.
8. Majdalani, N., Hernandez, D. and Gottesman, S. (2002) Regulation and mode of action of the second small RNA activator of RpoS translation, RprA. *Mol Microbiol*, **46**, 813-826.
9. Updegrove, T., Wilf, N., Sun, X. and Wartell, R.M. (2008) Effect of Hfq on RprA-rpoS mRNA pairing: Hfq-RNA binding and the influence of the 5' rpoS mRNA leader region. *Biochemistry*, **47**, 11184-11195.
10. Krin, E., Danchin, A. and Soutourina, O. (2010) RcsB plays a central role in H-NS-dependent regulation of motility and acid stress resistance in Escherichia coli. *Res Microbiol*, **161**, 363-371.
11. Rozanov, D.V., D'Ari, R. and Sineoky, S.P. (1998) RecA-independent pathways of lambdoid prophage induction in Escherichia coli. *J Bacteriol*, **180**, 6306-6315.
12. Sledjeski, D., Gottesman, S. (1995) A small RNA acts as an antisilencer of the H-NS-silenced *rcsA* gene of *Escherichia coli*. *Proc Natl Acad Sci U S A*, **92**, 2003-2007.
13. Ritzert, J.T., Minasov, G., Embry, R., Schipma, M.J. and Satchell, K.J.F. (2019) The Cyclic AMP Receptor Protein Regulates Quorum Sensing and Global Gene Expression in Yersinia pestis during Planktonic Growth and Growth in Biofilms. *mBio*, **10**.
14. Pannuri, A., Vakulskas, C.A., Zere, T., McGibbon, L.C., Edwards, A.N., Georgellis, D., Babitzke, P. and Romeo, T. (2016) Circuitry linking the catabolite repression and Csr global regulatory systems of *Escherichia coli*. *Journal of Bacteriology*, **198**, JB.00454-00416.
15. Cohen-Or, I., Shenhar, Y., Biran, D. and Ron, E.Z. (2010) CspC regulates rpoS transcript levels and complements hfq deletions. *Res Microbiol*, **161**, 694-700.
16. Park, H., McGibbon, L.C., Potts, A.H., Yakhnin, H., Romeo, T. and Babitzke, P. (2017) Translational Repression of the RpoS Antiadapter IraD by CsrA Is Mediated via Translational Coupling to a Short Upstream Open Reading Frame. *mBio*, **8**.
17. Mukherjee, A., Cui, Y., Ma, W., Liu, Y., Ishihama, A., Eisenstark, A. and Chatterjee, A.K. (1998) RpoS (sigma-S) controls expression of rsmA, a global regulator of secondary metabolites, harpin, and extracellular proteins in Erwinia carotovora. *J Bacteriol*, **180**, 3629-3634.
18. Chaparian, R.R., Tran, M.L.N., Miller Conrad, L.C., Rusch, D.B. and van Kessel, J.C. (2020) Global H-NS counter-silencing by LuxR activates quorum sensing gene expression. *Nucleic Acids Res*, **48**, 171-183.
19. Workman, R.E., Pammi, T., Nguyen, B.T.K., Graeff, L.W., Smith, E., Sebald, S.M., Stoltzfus, M.J., Euler, C.W. and Modell, J.W. (2021) A natural single-guide RNA repurposes Cas9 to autoregulate CRISPR-Cas expression. *Cell*.
20. Campa, A.R., Smith, L.M., Hampton, H.G., Sharma, S., Jackson, S.A., Bischler, T., Sharma, C.M. and Fineran, P.C. (2021) The Rsm (Csr) post-transcriptional regulatory pathway coordinately controls multiple CRISPR-Cas immune systems. *Nucleic Acids Res*, **49**, 9508-9525.

21. Patterson, A.G., Jackson, S.A., Taylor, C., Evans, G.B., Salmond, G.P.C., Przybilski, R., Staals, R.H.J. and Fineran, P.C. (2016) Quorum Sensing Controls Adaptive Immunity through the Regulation of Multiple CRISPR-Cas Systems. *Mol Cell*, **64**, 1102-1108.
22. Smith, L.M., Campa, A.R. and Fineran, P.C. (2022) In Marraffini, R. B. E. J. S. L. A. (ed.), *CRISPR: Biology and Applications*. ASM Press, pp. 145-162.
23. Hampton, H.G., Patterson, A.G., Chang, J.T., Taylor, C. and Fineran, P.C. (2019) GalK limits type I-F CRISPR-Cas expression in a CRP-dependent manner. *FEMS Microbiol Lett*.
24. Patterson, A.G., Chang, J.T., Taylor, C. and Fineran, P.C. (2015) Regulation of the Type I-F CRISPR-Cas system by CRP-cAMP and GalM controls spacer acquisition and interference. *Nucleic Acids Res*, **43**, 6038-6048.
25. Pul, U., Wurm, R., Arslan, Z., Geissen, R., Hofmann, N. and Wagner, R. (2010) Identification and characterization of *E. coli* CRISPR-cas promoters and their silencing by H-NS. *Mol Microbiol*, **75**, 1495-1512.
26. Arslan, Z., Stratmann, T., Wurm, R., Wagner, R., Schnetz, K. and Pul, U. (2013) RcsB-BglJ-mediated activation of Cascade operon does not induce the maturation of CRISPR RNAs in *E. coli* K12. *RNA Biol*, **10**, 708-715.
27. Hoyland-Kroghsbo, N.M., Paczkowski, J., Mukherjee, S., Broniewski, J., Westra, E., Bondy-Denomy, J. and Bassler, B.L. (2017) Quorum sensing controls the *Pseudomonas aeruginosa* CRISPR-Cas adaptive immune system. *Proc Natl Acad Sci U S A*, **114**, 131-135.
28. Thoma, S. and Schobert, M. (2009) An improved Escherichia coli donor strain for diparental mating. *FEMS Microbiol Lett*, **294**, 127-132.
29. Thomson, N.R., Crow, M.A., McGowan, S.J., Cox, A. and Salmond, G.P.C. (2000) Biosynthesis of carbapenem antibiotic and prodigiosin pigment in *Serratia* is under quorum sensing control. *Molecular Microbiology*, **36**, 539-556.
30. Hampton, H.G., Smith, L.M., Ferguson, S., Meaden, S., Jackson, S.A. and Fineran, P.C. (2020) Functional genomics reveals the toxin-antitoxin repertoire and AbiE activity in Serratia. *Microb Genom*, **6**.
31. Fineran, P.C., Slater, H., Everson, L., Hughes, K. and Salmond, G.P. (2005) Biosynthesis of tripyrrole and beta-lactam secondary metabolites in *Serratia*: integration of quorum sensing with multiple new regulatory components in the control of prodigiosin and carbapenem antibiotic production. *Mol Microbiol*, **56**, 1495-1517.
32. Wilf, N.M. and Salmond, G.P.C. (2012) The stationary phase sigma factor, RpoS, regulates the production of a carbapenem antibiotic, a bioactive prodigiosin and virulence in the enterobacterial pathogen Serratia sp. ATCC 39006. *Microbiology*, **158**, 648-658.
33. Smith, L.M., Jackson, S.A., Malone, L.M., Ussher, J.E., Gardner, P.P. and Fineran, P.C. (2021) The Rcs stress response inversely controls surface and CRISPR-Cas adaptive immunity to discriminate plasmids and phages. *Nat Microbiol*, **6**, 162-172.
34. Quintero-Yanes, A., Lee, C.M., Monson, R. and Salmond, G. (2020) The FloR master regulator controls flotation, virulence and antibiotic production in *Serratia* sp. ATCC 39006. *Environ Microbiol*, **22**, 2921-2938.
